# The mammalian decidual cell evolved from a cellular stress response

**DOI:** 10.1371/journal.pbio.2005594

**Published:** 2018-08-24

**Authors:** Eric M. Erkenbrack, Jamie D. Maziarz, Oliver W. Griffith, Cong Liang, Arun R. Chavan, Mauris C. Nnamani, Günter P. Wagner

**Affiliations:** 1 Department of Ecology and Evolutionary Biology, Yale University, New Haven, Connecticut, United States of America; 2 Systems Biology Institute, Yale University, West Haven, Connecticut, United States of America; 3 School of Biosciences, University of Melbourne, Melbourne, Australia; 4 Interdepartmental Program in Computational Biology and Bioinformatics, Yale University, New Haven, Connecticut, United States of America; 5 Department of Obstetrics, Gynecology, and Reproductive Science, Yale University Medical School, New Haven, Connecticut, United States of America; 6 Department of Obstetrics and Gynecology, Wayne State University, Detroit, Michigan, United States of America; University of Bath, United Kingdom of Great Britain and Northern Ireland

## Abstract

Among animal species, cell types vary greatly in terms of number and kind. The number of cell types found within an organism differs considerably between species, and cell type diversity is a significant contributor to differences in organismal structure and function. These observations suggest that cell type origination is a significant source of evolutionary novelty. The molecular mechanisms that result in the evolution of novel cell types, however, are poorly understood. Here, we show that a novel cell type of eutherians mammals, the decidual stromal cell (DSC), evolved by rewiring an ancestral cellular stress response. We isolated the precursor cell type of DSCs, endometrial stromal fibroblasts (ESFs), from the opossum *Monodelphis domestica*. We show that, in opossum ESFs, the majority of decidual core regulatory genes respond to decidualizing signals but do not regulate decidual effector genes. Rather, in opossum ESFs, decidual transcription factors function in apoptotic and oxidative stress response. We propose that rewiring of cellular stress responses was an important mechanism for the evolution of the eutherian decidual cell type.

## Author summary

Animals consist of a wide variety of cells that serve different functions depending on their location in the body. Cells with similar functions, or cell types, in different animal species are related both by an evolutionary line of descent—similar to the relatedness of species themselves—and by a developmental line of descent in the embryo. Networks of interacting genes, or gene regulatory networks, control gene expression in the cell, thereby specifying cell type identity. Understanding how new cell types arise by changing gene regulatory networks is critical both to comprehending fundamental aspects of human biology and to manipulating cell types in the laboratory. We approached this question by studying endometrial stromal fibroblast (ESF) cells from the uterus of humans and opossums, two distantly related mammals. We showed that the distantly related cell type in opossum expresses a similar set of regulatory genes as the human cell, but in response to pregnancy-related signals, the opossum cells induce a stress response. In the human cells, these signals induce differentiation into decidual cells, a specialized cell type present in humans and closely related mammals. These results suggest that a gene regulatory network that modulated an ancestral, pregnancy-related stress response was hijacked and repurposed to function in differentiation and specification of the decidual cell type.

## Introduction

Multicellular organisms consist of numerous specialized cells, or cell types, that play an important role in the structural and functional diversity of organisms. Evolutionary diversification of cell types in metazoans has been a significant source of novelty and was essential to the elaboration of increasingly complex body plans. One model that can explain the evolution of novel cell types is the “sister cell type” model, which suggests that cell types originate by differentiation from an ancestral cell type [1,2]. According to this model, novel cell types have arisen from ancestral cell types through modification of developmental programs leading to two derived cell types, termed “sister cell types.” The origination of novel cell types may allow for organisms to manage an imposed physiological or environmental challenge that may have induced stress or morbidity in the ancestral condition. However, while it is clear that cell types have diversified prodigiously in evolution, the molecular mechanisms leading to the origination of a novel cell type are not well understood [3].

The evolution of mammalian pregnancy offers an opportunity to investigate cell type origination. Intensive selective pressures during the evolution of mammalian pregnancy led to the evolution of many functional specializations of the uterus that accommodate the implantation of the embryo and development of the placenta, including proper control of an ancestral implantation-induced inflammatory response [4,5]. These novelties include the origin of specialized cell types such as the decidual stromal cell (DSC), the uterine natural killer cell, and a specialized form of resident macrophages [6].

During the menstrual cycle and pregnancy, human decidual stromal cells (HsDSCs) differentiate from endometrial stromal fibroblasts (HsESFs) on exposure to progesterone and signals from the embryo [7]. Responding to these signals, genes critical to human DSC differentiation drive and install a complex decidualization gene regulatory network (GRN). Numerous transcription factors have been shown to transcriptionally and post-translationally interact to regulate effector gene sets conferring DSC cell type identity. Phylogenetic cell type studies make clear that eutherian endometrial stromal fibroblast (ESF) and DSC are sister cell types [8]. While ESFs are found in the oviduct of numerous amniotes, DSCs are exclusive to eutherians [9]. Moreover, it is clear that DSCs evolved from an ancestral ESF cell type, hereafter referred to as paleo-ESF, i.e., ESF that cannot give rise to DSC and are not derived from cells that can. This cell type existed prior to the stem lineage of eutherian mammals, having diverged 65 to 80 million years ago [10], i.e., DSC evolved after the most recent common ancestor of marsupials and eutherians and prior to the most recent common ancestor of eutherian mammals [11]. Hence, the evolutionary origin of DSC is an outstanding model to investigate the molecular mechanisms that led to the origin of a novel cell type.

To characterize the molecular changes that gave rise to the origin of the decidual stromal cell type, we isolated ESFs of the marsupial grey short-tailed opossum *Monodelphis domestica*, hereafter called MdESFs, which we use as a proxy for paleo-ESF. In humans and other eutherians, neo-ESF differentiates into DSC in utero when exposed to progesterone and estrogen, as well as ligands upstream of cyclic AMP (cAMP)/protein kinase A (PKA) signaling such as prostaglandin E2 (PGE2) [12–14] and relaxin (RLN) [15]. We assayed the response of MdESF to the stimuli that differentiate HsESF to HsDSC in vitro in order to identify the ancestral gene regulatory program from which the core network of DSC evolved. We found, surprisingly, that core components of the decidual GRN are responsive to progesterone and cAMP in opossum ESF, but rather than undergoing DSC differentiation, these genes regulate a cellular stress response.

## Results

### ESF isolation from *M*. *domestica*

We utilized an established protocol to isolate MdESF by Percoll column gradient [16]. We validated by immunostaining and western blotting that cells isolated by this procedure are positive for the mesenchymal marker vimentin and negative for the epithelial marker cytokeratin (S1 Fig). Relative to other layers in the column, these cells expressed higher levels of the ESF markers *HOXA11*, *HOXA10*, and *PGR*. We also show that these cell preparations have low levels of *CD45*, a marker of white blood cells, compared to RNA isolated from opossum spleen (S1 Table).

### 8-br-cAMP/medroxyprogesterone acetate induce decidual regulatory genes

**Fig 1:**
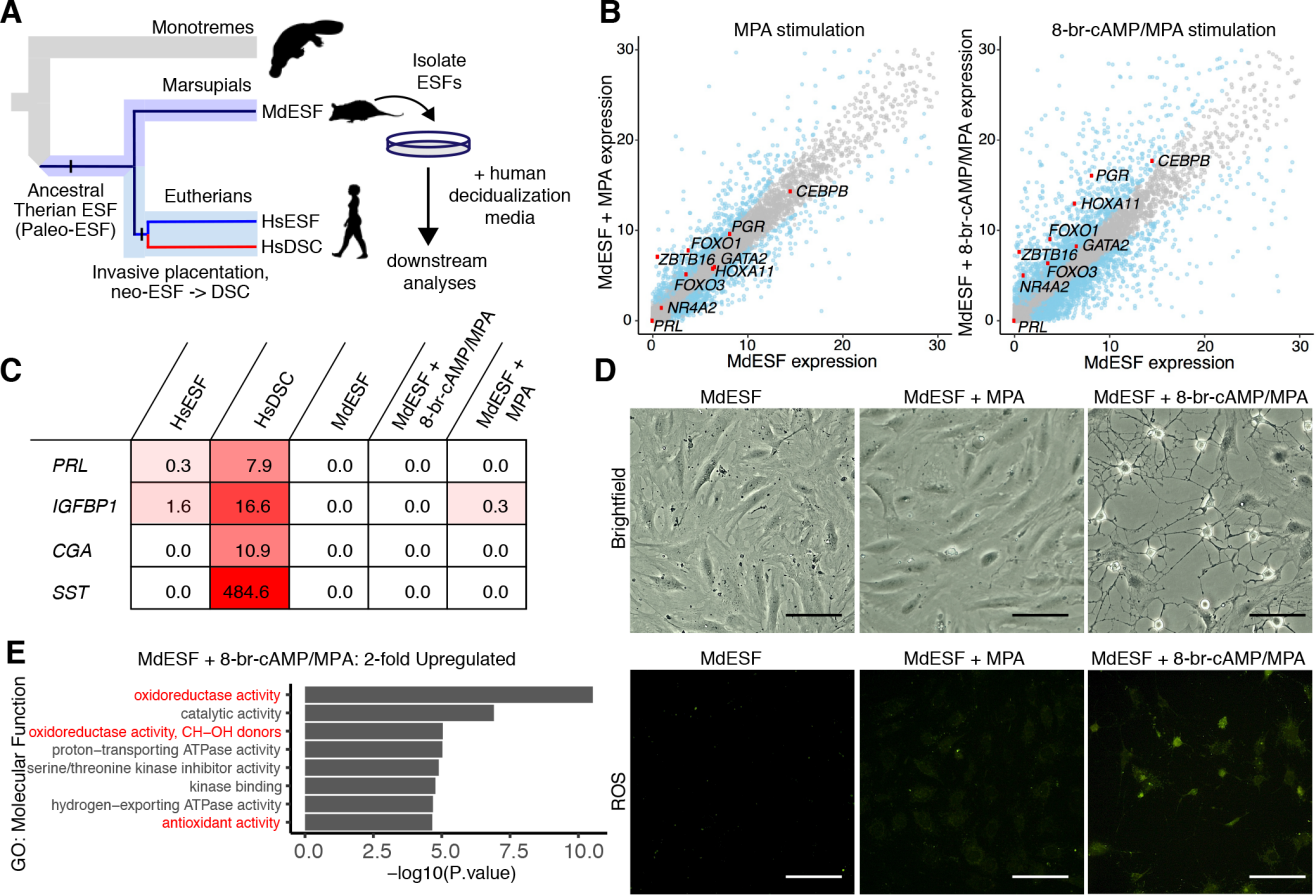
Transcriptomic and morphological response of MdESF to eutherian decidualizing stimuli. (A) Experimental strategy and cell type phylogeny of mammalian ESF cell types. ESFs in crown group marsupials and placentals are homologous and descend from a common ancestral therian ESF. In eutherians, DSCs arose from ancestral eutherian ESFs and thus is a sister cell type to eutherian ESFs. (B) Transcriptional response of core regulatory genes involved in eutherian decidualization in MdESF treated for 2 days with either MPA or decidualizing stimuli. Blue dots represent significant differential expression relative to unstimulated MdESF (*n* = 3, *p* < 10^−6^). Grey dots represent no significant change in expression. Each point represents the mean of three replicates. (C) Gene expression heatmap (mean of three replicates in TPM) of classical decidual markers in HsESF, HsDSC, and MdESF treated with decidualizing stimuli for 2 days. Mean of three replicates is shown. (D) Morphological response of and ROS detection in unstimulated MdESF and MdESF stimulated with either MPA or decidualizing stimuli for 3 days. ROS detection by treatment with H2DCFDA, a general oxidative stress indicator. Scale bars are 10 μm. (E) Differential expression of up-regulated GO categories in MdESF treated with decidualizing stimuli for 2 days relative to unstimulated control (*n* = 3, adjusted *P* values for each GO term are shown). Terms related to oxidative stress are indicated in red. Underlying data are provided in S1 Data (Fig 1E) and S2 Data (Fig 1B). Human transcriptome data were previously published in Kin and colleagues [8]. *Unedited silhouettes of a monotreme and a marsupial are credited to Sarah Werning and are used under license http://creativecommons.org/licenses/by/3.0/*. DSC, decidual stromal cell; ESF, endometrial stromal fibroblast; GO, gene ontology; H2DCFDA, 2′,7′ dichlorodihydrofluorescein diacetate; HsDSC, human decidual stromal cell; HsESF, human endometrial stromal fibroblast; MPA, medroxyprogesterone acetate; ROS, reactive oxygen species; TPM, transcripts per million

We assayed the response of MdESF to treatment with eutherian ESF differentiation media containing the cAMP analogue 8-br-cAMP and the progesterone analogue medroxyprogesterone acetate (MPA) (Fig 1A), hereafter referred to as decidualizing stimuli or 8-br-cAMP/MPA. RNA sequencing (RNAseq) of both stimulated and unstimulated MdESF revealed endogenous expression of numerous core regulatory genes critical to eutherian decidualization (Fig 1B). From a curated list of 28 transcription factor (TF) genes with documented roles in decidualization (Table 1), 22 are expressed in stimulated MdESF and 13 are significantly up-regulated (*p* < 0.05) (Fig 1B and S2A Fig). Seven decidualization TF genes are down-regulated, though still expressed, and two TFs are unchanged in expression. Most notably, the up-regulated gene set contains numerous TFs with well-characterized roles in decidualization: *FOXO1* [17,18], *PGR* [19], *CEBPB* [17,20], *HOXA10* [21,22], *HOXA11* [23,24], *GATA2* [25], *ZBTB16* [26,27], *KLF9* [28–30], *HAND2* [31], *STAT3* [32,33], and *MEIS1* [34] (Fig 1B and S2A Fig). In contrast to this conserved transcriptional regulatory response, classical markers of decidualization, e.g., *PRL*, *IGFBP1*, *CGA*, and *SST*, are neither expressed in unstimulated MdESF nor induced in response to decidualizing stimuli (Fig 1C). We conclude that a substantial part of the DSC core GRN is also in place in opossum ESF and is responsive to progesterone and cAMP but does not control a decidual phenotype.

**Table 1:** List of 28 TFs with documented roles in decidualization with references.

TF	Reference(s)
*CEBPB*	[17,20]
*E2F8*	[35]
*EBF4*	[8]
*ETS1*	[36]
*FOS*	[37]
*FOXM1*	[38]
*FOXO1*	[18]
*GATA2*	[25]
*HAND2*	[31]
*HOXA10*	[21,22]
*HOXA11*	[23,24]
*HOXD9*	[16]
*HOXD11*	[39]
*HOXD12*	[16]
*KLF9*	[28–30]
*MEIS1*	[34]
*NR2F2*	[40]
*PBX2*	[41]
*PGR*	[19]
*PRRX2*	[42]
*RUNX1*	[43]
*STAT3*	[32,33]
*STAT5B*	[44]
*TFAP2C*	[16]
*TWIST1*	[45]
*WT1*	[46]
*ZBTB16*	[26,27]

**Abbreviation:** TF, transcription factor.

We conducted experiments to determine if the observed up-regulation of regulatory genes critical to eutherian decidualization is specific to both the progesterone receptor as well as specific to the *M*. *domestica* ESF cell type. As MPA can also stimulate the glucocorticoid receptor (GR), we knocked down *GR* with small interfering RNA (siRNA) and subsequently assayed the transcriptional response of MPA-responsive regulatory genes in MPA-stimulated MdESF. We observed no significant change in RNA abundance for five decidualization regulatory genes (S2B Fig), suggesting that the observed up-regulation of these factors in response to MPA is not associated with stimulation of GR.

It could also be argued that the observed response is a more general feature of *M*. *domestica* fibroblasts rather than specific to MdESF. Thus, we sought to determine if up-regulation of decidualization regulatory genes is specific to the *M*. *domestica* ESF cell type or whether a similar response also occurs in skin fibroblasts from *M*. *domestica*. We isolated skin fibroblasts from *M*. *domestica*, stimulated them with either 8-br-cAMP/MPA or PGE2/MPA, and assayed six decidualization TFs by qPCR. Our results showed a strong up-regulation of *ZBTB16* in response to 3-day treatment of either 8-br-cAMP/MPA or PGE2/MPA (S2C Fig). Conversely, five other TFs that were up-regulated in MdESF in response to either treatment were unchanged in RNA abundance or were down-regulated. This result suggests that the induction of decidual regulatory genes is specific to endometrial fibroblasts in the opossum and, interestingly, that *ZBTB16* may be a more general inducible factor in fibroblasts with elevated intracellular levels of cyclic AMP.

### 8-br-cAMP/MPA induce cell stress in MdESF

**Fig 2:**
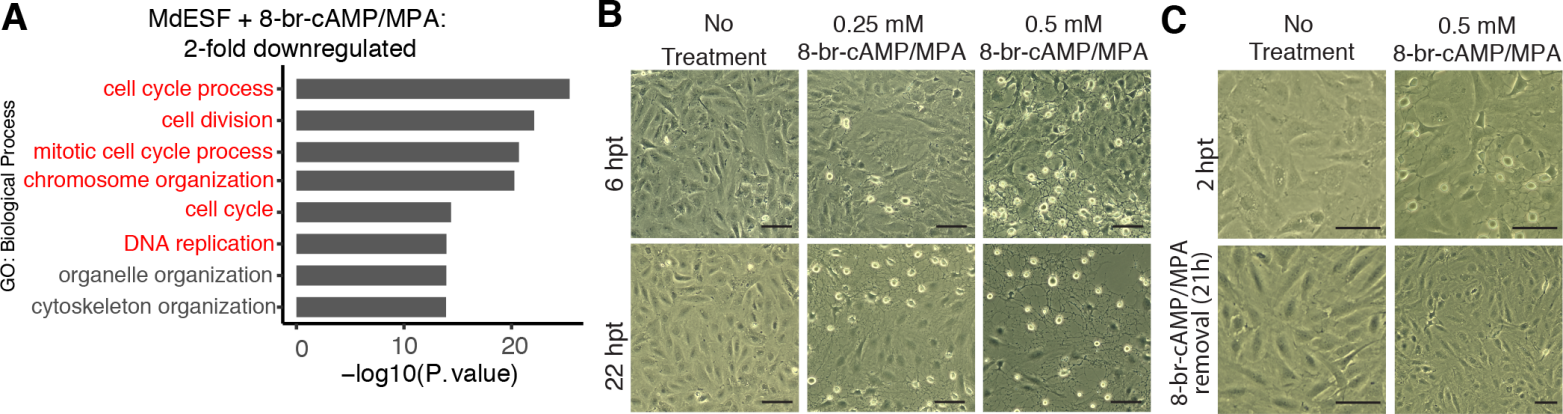
Transcriptional, cAMP-concentration dependent, and reversible response of MdESF to decidualizing stimuli. (A) GO categories of down-regulated genes in MdESF after treatment with decidualizing stimuli for 2 days relative to unstimulated control (*n* = 3, *p* < 10^−6^). Terms related to cell cycle are indicated in red. (B) Concentration-dependent morphological response of MdESF to 8-br-cAMP/MPA treatment after 6 hours (top panels) and 22 hours (bottom panels). (C) MdESF morphological response to 8-br-cAMP/MPA treatment is reversible after 1 day in growth media. Images were taken 2 hours after treatment, at which time media with decidualizing stimuli were changed to growth media. Images were subsequently acquired after 19 hours in growth media. Scale bars are 10 μm. Underlying data for GO analysis are provided in S1 Data. cAMP, cyclic AMP; GO, gene ontology; MPA, medroxyprogesterone acetate

Gene ontology (GO) enrichment analysis of differentially expressed genes after 8-br-cAMP/MPA treatment revealed up-regulation of genes associated with oxidative stress, mitochondrial stress, and apoptosis, as well as down-regulation of genes associated with mitosis, DNA replication, and cytoskeletal organization (Fig 1E, Fig 2A). Outwardly, stimulated MdESF exhibited a rapid morphological response suggestive of cytoplasmic architectural remodeling (Fig 1D, Fig 2B, S1 Movie). The extent of this morphological response was dependent on both 8-br-cAMP concentration and duration of treatment (Fig 2B, S3A Fig). Remarkably, this morphological effect was reversible insofar as the cells reverted back to their normal morphology within 19 hours after withdrawal of decidualizing stimuli (Fig 2C, S3B Fig). GO treemaps, which represent the function of genes and degree of their differential expression in response to 8-br-cAMP/MPA, supported the hypothesis that stimulated MdESF undergo a cellular stress response, as GO terms associated with endoplasmic reticulum (ER) stress, apoptosis, reactive oxygen species (ROS) metabolism, and protein folding response were significantly up-regulated (S2D Fig). In line with this observation, stimulated MdESF exhibited elevated levels of intracellular ROS relative to unstimulated cells or cells stimulated with MPA alone (Fig 1D, S3C Fig). These data indicate that treating MdESF with decidualizing stimuli results in a rapid morphological response that is associated with increased intracellular ROS and the induction of genes counteracting oxidative stress, suggesting that, rather than leading to decidual differentiation, MdESF exposed to decidualizing stimuli undergo a classical cellular stress response.

### PGE2 is likely a natural ligand of ESFs

**Fig 3:**
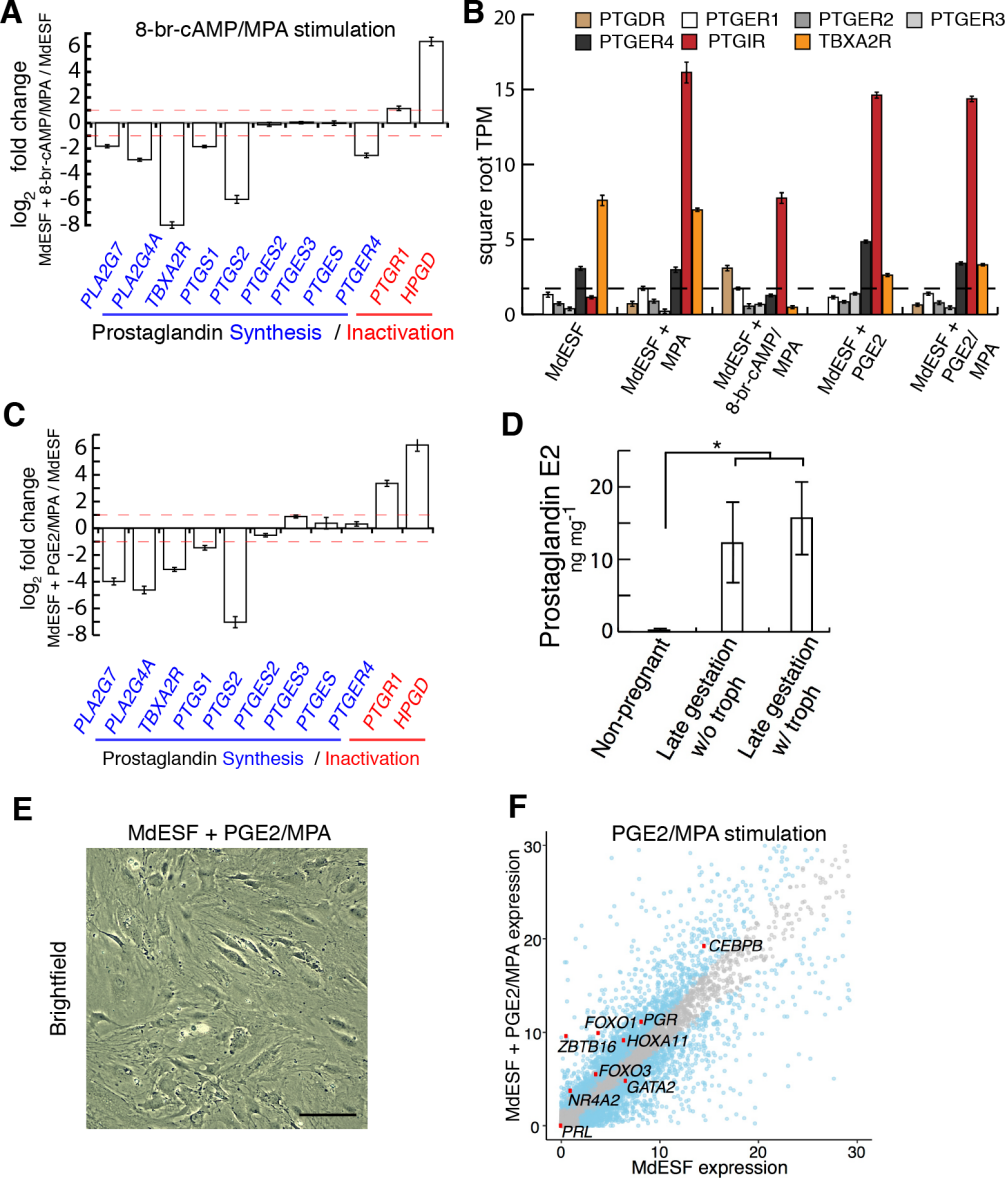
Transcriptomic and morphological response of MdESF to MPA and PGE2. (A) Transcriptional response of KEGG pathway genes associated with prostaglandin signaling in MdESF treated with decidualizing stimuli for 2 days relative to unstimulated control (*n* = 3, log2 fold change shown). Red dashed line represents 2-fold change. (B) Expression of orthologous receptors in MdESF for prostaglandin signaling pathways in unstimulated MdESF or stimulated for 2 days with MPA alone, 8-br-cAMP/MPA, PGE2 alone, or PGE2/MPA. Average square root TPM of three replicates is shown. Error bars show standard error of the mean. (C) Transcriptional response of prostaglandin synthesis and inactivation genes in MdESF treated for 2 days with PGE2/MPA. Fold change relative to unstimulated control is shown (*n* = 3, log2 fold change shown). Red dashed line represents 2-fold change. (D) Increased PGE2 (in ng per mg total protein) in pregnant (late gestation, 13.5 days) versus nonpregnant *M*. *domestica* females (*n* = 2 females per sample) as measured by ELISA (*, two-tailed *t* test on log transformed data, *p* = 0.014). (E) MdESF treated for 2 days with PGE2/MPA do not exhibit the dendritic phenotype. Scale bars are 10 μm. (F) Transcriptional response of core regulatory genes involved in eutherian decidualization in MdESF treated with PGE2/MPA for 2 days. Blue dots represent significant differential expression relative to unstimulated MdESF (*n* = 3, *p* < 10^−6^). Grey dots represent no significant change in expression. Each point represents the mean of three replicates. Underlying data are provided in S1 Data (Fig 3A–3D) and S2 Data (Fig 3F). KEGG, Kyoto Encyclopedia of Genes and Genomes; MPA, medroxyprogesterone acetate; PGE2, prostaglandin E2; TPM, transcripts per million.

Next, we considered whether stress induced by treatment with decidualizing stimuli could be an artifact of treating cells with extracellular 8-br-cAMP, rather than a natural ligand activating intracellular cAMP signaling. To address this, we sought a physiologically relevant signal that increases intracellular cAMP in these cells. PGE2 signaling is of particular interest given that (1) PGE2 is able to induce decidualization via cAMP signaling in human and rodent ESFs [12,14], (2) the PGE2 receptor *PTGER4* is widely expressed in ESFs in mammals [16], and (3) the recent finding that prostaglandin synthase (PTGS, also known as “COX2”) and prostaglandin E synthase (PTGES) are both expressed in the opossum uterus after embryo attachment [5]. Furthermore, PGE2 is likely a key component of the inflammatory signaling from which the eutherian implantation reaction is derived [5,6,47]. In our 8-br-cAMP/MPA stimulated cells, we see a particularly striking effect on lipid metabolism, a critical pathway in the production of phospholipid-derived prostaglandins (S2D Fig). Indeed, prostaglandin Kyoto Encyclopedia of Genes and Genomes (KEGG) pathway genes were enriched in lipid metabolism, e.g., 33% of genes listed in fatty-acid derivative metabolic process are involved in prostaglandin metabolism (S2D Fig). Furthermore, transcriptomic analyses of stimulated MdESF suggested that 8-br-cAMP/MPA treatment negatively regulates the predominant PGE2 receptor, *PTGER4*, as well as genes for synthesis of prostaglandins, e.g., *PTGS2* and *PTGES* (Fig 3A), and positively regulates catabolic enzymes that function to degrade prostaglandins, e.g., *HPGD* and *PTGR1* (Fig 3A). These data suggest stimulated MdESFs compensate for the effect of 8-br-cAMP by modulating the prostaglandin synthesis and signaling pathways, further suggesting that PGE2 is likely the natural ligand of MdESF activating the cAMP/PKA pathway.

A survey of RNAs present in unstimulated MdESF showed that only two prostaglandin signaling receptors, *PTGER4* and *TBXA2R*, are expressed in these cells (Fig 3B). In order to test whether PGE2 could be the natural ligand inducing intracellular cAMP signaling in these cells, we assayed by RNAseq the response of MdESF to PGE2 with and without MPA. KEGG pathway genes involved in prostaglandin synthesis and inactivation exhibited similar differential regulation in response to PGE2/MPA as do decidualizing stimuli, suggesting similar regulatory responses by MdESF (Fig 3C). Interestingly, a survey of prostaglandin signaling components revealed a strong up-regulation of the prostacyclin receptor (*PTGIR*) across all treatment groups (Fig 3B). Uterine tissue from pregnant and nonpregnant *M*. *domestica* showed substantially higher amounts of PGE2 in pregnant females versus nonpregnant females, suggesting that PGE2 increases in utero during gestation (Fig 3D). Contrary to treatment with decidualizing stimuli, MdESF treated with PGE2 and with or without MPA did not exhibit a readily apparent dendritic phenotype (Fig 3E, S4A Fig). Nevertheless, PGE2/MPA-treated MdESF do show elevated levels of intracellular ROS (S4B Fig).

We next investigated the effect of PGE2/MPA on the expression of core decidualization regulatory genes. Remarkably, all 22 decidual TF regulatory genes expressed in 8-br-cAMP/MPA–stimulated MdESF cells are also expressed in PGE2/MPA-stimulated cells (Fig 3F, S2A Fig), showing a marked nonparametric correlation in expression levels (Spearman’s rho = 0.863, *p* = 3.46 × 10^−9^), and 12 of the 13 up-regulated genes under 8-br-cAMP/MPA are also up-regulated with PGE2/MPA. However, this up-regulation was not seen when MdESFs were treated with PGE2 alone (S4C Fig), indicating a synergistic effect of elevated intracellular cAMP and components responding to MPA. Similar to the response to MPA alone and 8-br-cAMP/MPA, previously characterized marker genes of human decidualization did not respond to 2-day treatment with either PGE2 or PGE2/MPA (Table 2). We conclude that responding to PGE2 signaling is a physiological part of MdESF biology and that PGE2/MPA also regulates the expression of the same TF network as 8-br-cAMP/MPA treatment. Moreover, the results suggest that this PGE2/MPA-induced TF network is homologous to that activated during the differentiation of human DSCs.

**Table 2:** Response of decidualization marker genes in MdESF treated with either PGE2 or PGE2/MPA. Mean TPM of three replicates is shown.

Gene/Treatment	PGE2	PGE2/MPA
*PRL*	0.04	0.00
*IGFBP1*	0.00	0.00
*CGA*	0.00	0.00
*SST*	0.00	0.00

**Abbreviations:** PGE2, prostaglandin E2; MPA, medroxyprogesterone acetate; TPM, transcripts per million.

### PGE2-induced stress response in MdESF

**Fig 4:**
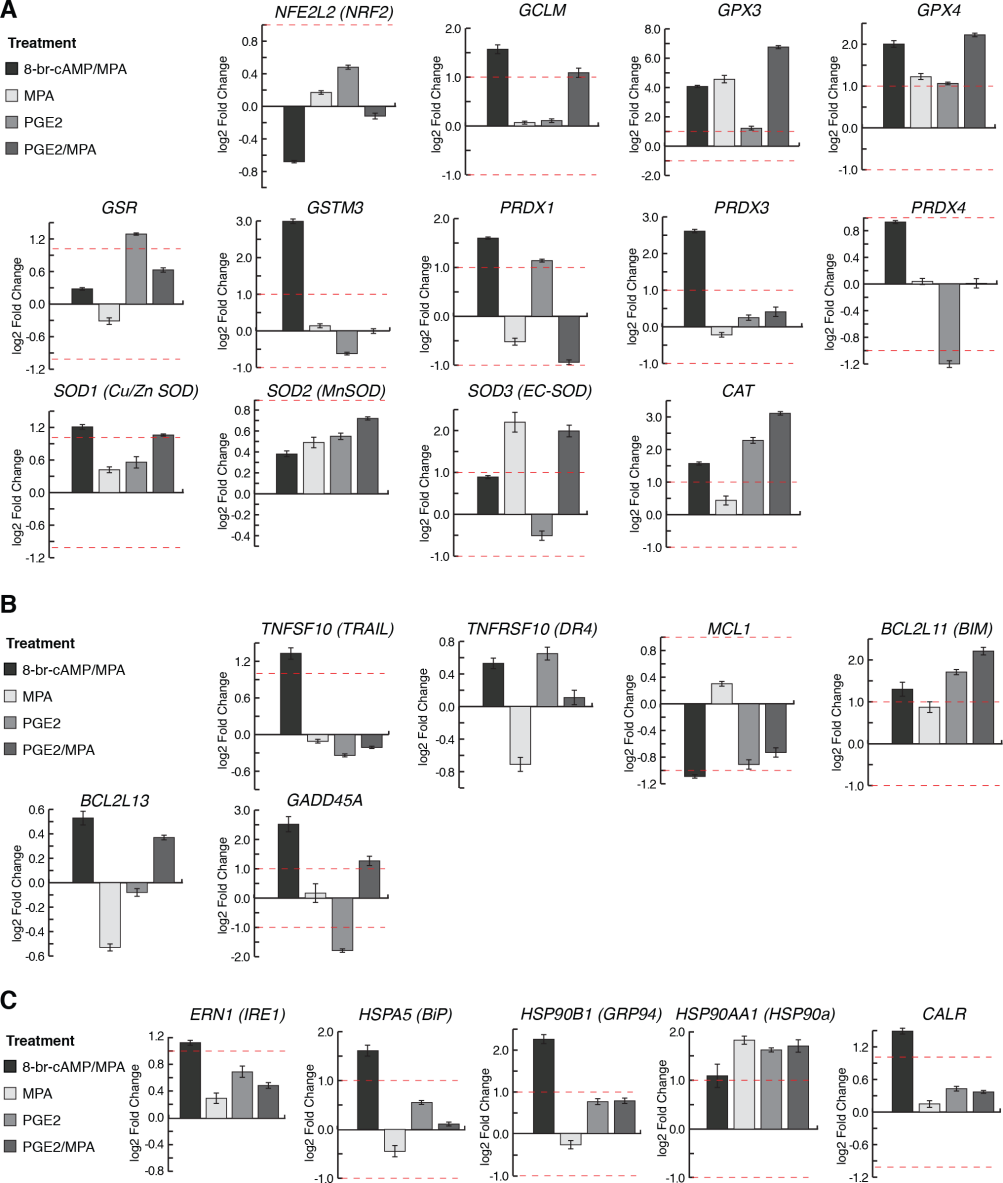
Stress-related gene expression changes in all MdESF 2-day treatments relative to unstimulated control. Genes of interest are shown that are known to be directly involved in (A) oxidative stress response; (B) apoptosis; (C) unfolded protein response in the ER due to oxidative stress. Underlying data are provided in S1 Data. ER, endoplasmic reticulum.

We next asked if, as seen in cells treated with decidualizing stimuli, PGE2/MPA treatment also induced a stress response in MdESF. We surveyed differential expression of genes involved in oxidative stress response, apoptosis, and ROS-associated ER stress (unfolded protein response [UPR]) (Fig 4). MdESF treated with either 8-br-cAMP/MPA or PGE2/MPA significantly up-regulated genes associated with counteracting oxidative stress, including *GCLM*, *GPX3*, *GPX4*, *SOD1*, *SOD3*, and *CAT* (Fig 4A). Both treatments up-regulated the apoptotic genes *BCL2L11 (BIM)* and *GADD45A* (Fig 4B). In contrast to treatment with PGE2/MPA, cAMP/MPA induced a distinct stress response in genes associated with UPR and TNF-related apoptosis-inducing ligand (TRAIL)-related apoptosis, up-regulating *ERN1 (IRE1)*, *HSPA5*, *HSP90B1*, *HSP90AA1*, *CALR*, and *TNFSF10* (Fig 4B and 4C). Lastly, to determine if clusters of genes associated with oxidative stress and apoptosis were differentially expressed in both cAMP/MPA and PGE/MPA, we analyzed GO term clusters shared between the treatments. This analysis also suggested a shared stress response in MdESF treated with decidualizing stimuli or PGE2/MPA, in which GO terms associated with stress and inflammation, e.g., “regulation of reactive oxygen species metabolism,” “protein folding,” and “leukocyte degranulation,” were shared between these treatments (S2D Fig, S5 Fig). Similarly, PGE2 alone and PGE2/MPA shared GO terms specifically related to “hypoxia,” “autophagy,” and “regulation of cell death” (S5 Fig, S6 Fig). These results suggest that PGE2/MPA, signals that are present in the pregnant opossum uterus, induce a stress response similar to treatment with 8-br-cAMP/MPA, including elevated levels of intracellular ROS (one-tailed *t* test on log transformed data, *p* = 0.0186), but without the dendritic morphological response (Fig 3E, S4B Fig). We conclude that the in vitro 8-br-cAMP/MPA–induced stress reaction is mimicked with the more physiological PGE2/MPA treatment. Furthermore, these data are consistent with a conserved role for PGE2 during pregnancy of therian mammals.

### Subcellular localization of FOXO1

**Fig 5:**
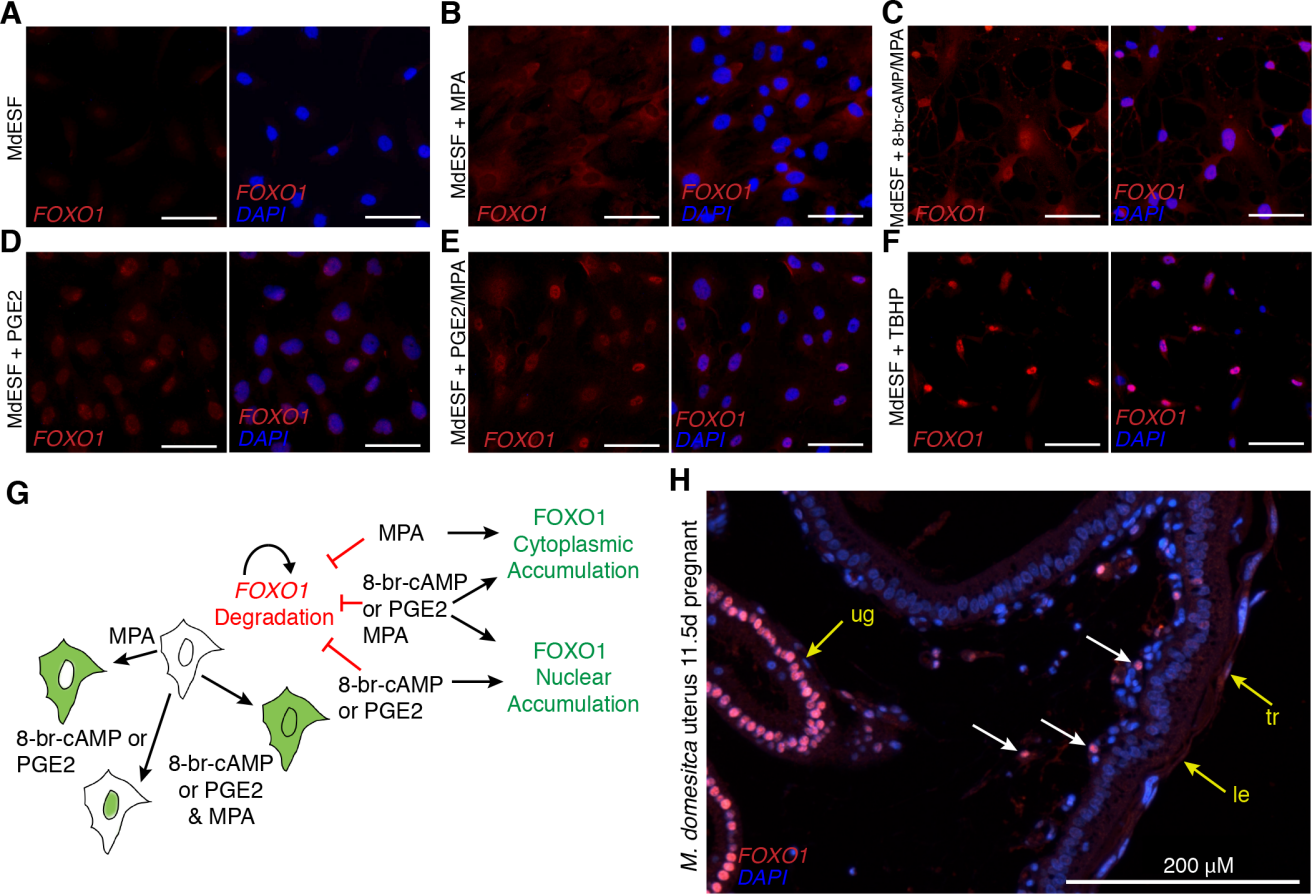
Post-translational regulatory control of FOXO1 by PGE2 and oxidative stress in MdESF. (A-H) Immunofluorescence of FOXO1 in MdESF and pregnant *M*. *dometica* uterus. (A) FOXO1 is not detected above background in unstimulated MdESF. (B) FOXO1 protein is detected in the cytoplasm but not in the nucleus in MdESF treated for 2 days with MPA alone. (C) FOXO1 translocates to the nucleus and is detected in the cytoplasm in MdESF treated with decidualzing stimuli 8-br-cAMP/MPA for 2 days. (D-E) FOXO1 is detected in the nucleus and cytoplasm in MdESF treated for 2 days with either PGE2 alone or PGE2/MPA. (F) Oxidative stress induces FOXO1 to translocate to the nucleus in MdESF treated with tert-butyl hydrogen peroxide TBHP for 2 hours. Scale bars are 10 μm. (G) Model showing post-translational regulatory control of FOXO1 in MdESF treated for 2 days with stimuli in this study. (H) FOXO1 immunofluorescence of uterine tissue in cross section during late gestation (11.5 d.p.c.). All samples counterstained with DAPI. Arrows indicate FOXO1 detection. cAMP, cyclic AMP; d.p.c., days post coitus; FOXO, forkhead box class O; le, luminal epithelium; MPA, medroxyprogesterone acetate; PGE2, prostaglandin E2; ug, uterine glands; TBHP, tert-butyl hydrogen peroxide; tr, trophoblast.

In many cells, forkhead box class O (FOXO) TF family members generically function in stress response, counteracting oxidative stress, and apoptosis, as well as regulating gluconeogenesis and glycolysis [48]. FOXO1 also is an early acting TF in the differentiation of human DSCs [49]. Therefore, we sought to compare how *FOXO1* mRNA and FOXO1 protein stability and subcellular localization are regulated in opossum ESFs. As observed in human ESFs, *FOXO1* RNA is present in unstimulated MdESF, but FOXO1 protein is absent [16,50], likely due to protein kinase B (Akt)-dependent proteasomic degradation [48] (Fig 5A). In response to MPA treatment, FOXO1 accumulates in the cytoplasm (Fig 5B), suggesting MPA alone can counteract the proteasomic FOXO1 degradation. Decidualizing stimuli and treatment with PGE2/MPA resulted in nuclear translocation of FOXO1 as well as cytoplasmic loading (Fig 5C–5E), suggesting cAMP/PKA signaling controls FOXO1 nuclear localization. In response to induction of oxidative stress, FOXO1 behaved similarly to cAMP or PGE2 treatment, suggesting that post-translational modifications of FOXO1 act as a sensor of oxidative stress in MdESF (Fig 5F). Moreover, immunofluorescence on uterine sections from pregnant *M*. *domestica* females in the late stages of gestation (11.5 days post coitus [d.p.c.]) found nuclear FOXO1 in uterine stromal cells near the luminal epithelium, suggesting that FOXO1 activation is part of the physiological role of MdESF during pregnancy (Fig 5H). Strikingly similar results were obtained for FOXO1 in human DSCs (S7A Fig), suggesting post-translational regulatory control of FOXO1 as found in human DSCs in response to decidualizing stimuli has been inherited from the ancestral paleo-ESF and ancestrally was part of a PGE2-induced cell stress response (Fig 5G).

### FOXO counteracts apoptosis in opossum ESF

**Fig 6:**
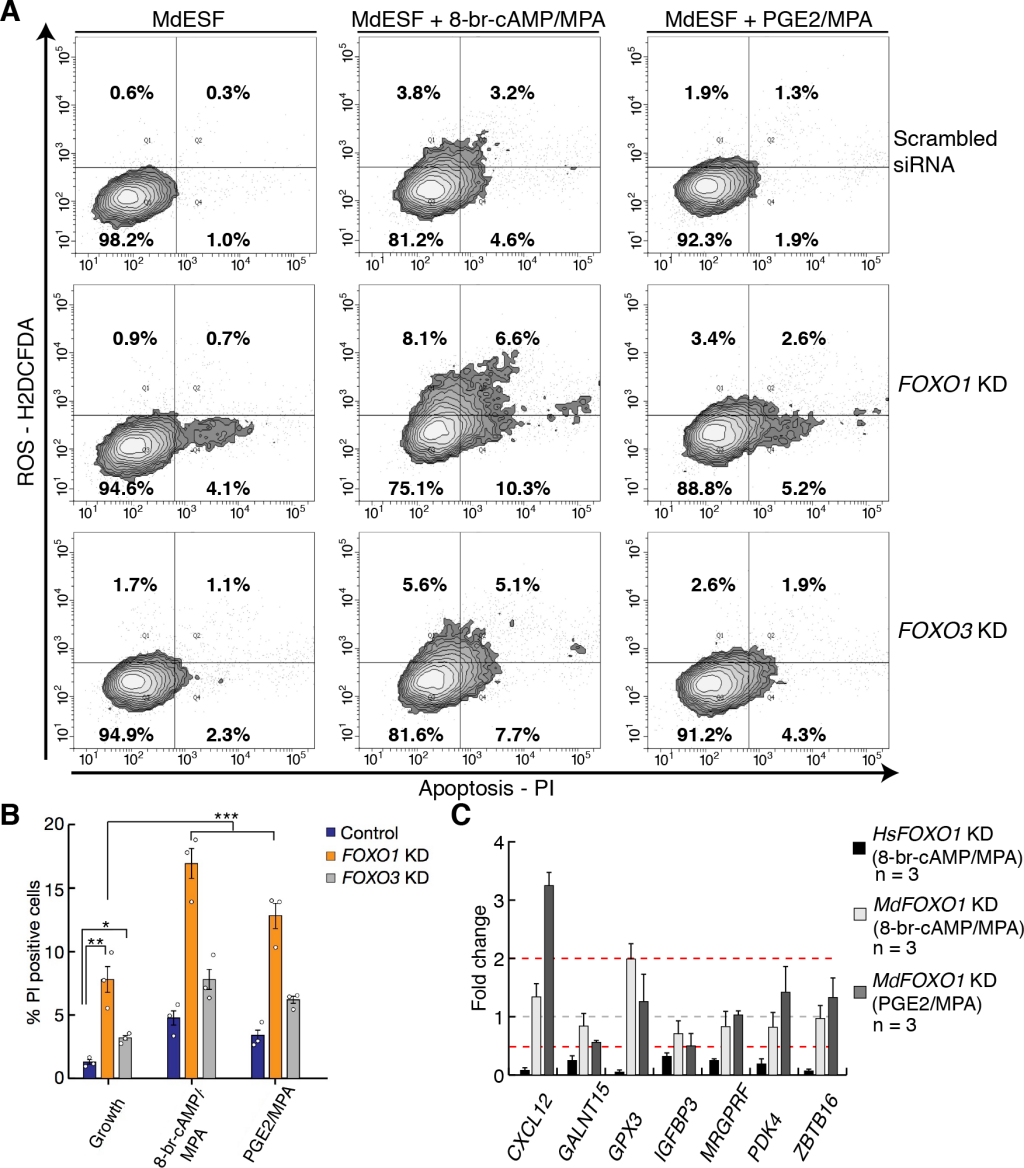
FOXO proteins counteract apoptosis in stimulated MdESF. (A) Treatment of MdESF with 8-br-cAMP/MPA or PGE2/MPA for 3 days results in more cells positive for ROS and apoptosis, PI staining (top panels). KD of *FOXO1* in unstimulated MdESF increases cell counts for apoptotic cells relative to control treated with scrambled siRNA control (middle, left panel). KD of *FOXO1* and treatment with cAMP/MPA or PGE2/MPA for 3 days significantly increased cell counts for apoptosis along with a moderate increase in ROS (middle panels). KD of *FOXO3* in unstimulated MdESF increases cell counts for ROS and apoptosis (bottom, left panel). KD of *FOXO3* and subsequent 3-day treatment with decidualizing stimuli or PGE2/MPA results in increased cell counts for ROS and apoptosis (bottom panels). Quadrants are set to maximum extent of boundaries in unstimulated MdESF treated with scrambled siRNA. Q1, positive for ROS only; Q2, positive for ROS and PI; Q3, negative for ROS and PI; Q4, negative for ROS and positive for PI. Percentage in each quadrant represents mean of three replicates. (B) Bar graph showing mean percent positive cells for quadrants 2 (ROS and PI positive) and 4 (PI positive only) (*n* = 3, 2-tailed *t* test, * *p* < 0.05, ** *p* < 0.005; ANOVA on log transformed fraction of cells, *** *p* = 5.91 × 10^−8^**).** Blue, scrambled siRNA control; orange, *FOXO1* KD; gray, *FOXO3* KD. Empty circles represent individual data points for each replicate. (C) Fold change of *FOXO1*-responsive genes in human DSCs in MdESF stimulated for 3 days with either 8-br-cAMP/MPA or PGE2/MPA in *FOXO1* perturbation background relative to stimulated control treated with scrambled siRNA. Data are the average of three replicates ± standard error of the mean. Underlying data are provided in S1 Data. cAMP, cyclic AMP; DSC, decidual stromal cell; FOXO, forkhead box class O; KD, knockdown; MPA, medroxyprogesterone acetate; PGE2, prostaglandin E2; PI, propidium iodide; ROS, reactive oxygen species; siRNA, small interfering RNA.

In order to assess the functional role of FOXO1 activation in opossum ESF, we assayed oxidative stress and apoptosis by treatment with 2′,7′ dichlorodihydrofluorescein diacetate (H2DCFDA) (to detect ROS) and propidium iodide (PI) (to detect early stages of apoptosis) in stimulated and unstimulated MdESF (Fig 6). Apoptosis, i.e., PI staining, was markedly elevated in MdESF treated with decidualizing stimuli (Fig 6A and 6B; 4-fold increase, one-tailed *t* test *p* = 2.0 10^−5^). That was also the case for PGE2/MPA but to a lesser degree (1.8-fold increase, one-tailed *t* test *p* = 0.015). To determine if FOXO TFs function in this stress response, we transfected MdESF with siRNAs targeting *FOXO1* and *FOXO3* RNA transcripts and subsequently treated them with 8-br-cAMP/MPA or PGE2/MPA. We confirmed depletion of *FOXO1* RNA (as well as *FOXO3* RNA) by qPCR and depletion of FOXO1 protein by western blot (S7B Fig, S7C Fig). siRNA-mediated knockdown (KD) of *FOXO1* and *FOXO3* increased signals for apoptosis (Fig 6A and 6B) (ANOVA on log transformed fluorescence values, KD effects *FOXO1* = 2.4-fold, *FOXO3* = 1.8-fold, overall ANOVA *p* = 5.91 10^−8^). Surprisingly, we did not find a significant interaction effect of *FOXO1* and *FOXO3* KD, suggesting they function additively in protecting against apoptosis (Fig 6A and 6B). However, there is no significant effect of *FOXO* KD on ROS levels in our data, suggesting that ROS production in response to decidualizing stimuli is not regulated by FOXO proteins.

*FOXO1* KD resulted in marginally more cells positive for apoptosis than did *FOXO3* perturbation (Fig 6B; 1.31-fold, *p* = 0.045). These results suggest that the ancestral function of FOXO genes in paleo-ESF was similar to the highly conserved, pan-metazoan roles of FOXO genes in classical stress response [51]. Therefore, we hypothesized that the regulatory linkages downstream of FOXO1 have diverged since the eutherian–metatherian split. From a list of genes in human DSCs that are positively regulated by FOXO1 (i.e., decrease in expression after *FOXO1* KD and treatment with decidualizing stimuli), we selected seven genes that are also strongly up-regulated in MdESF stimulated with 8-br-cAMP/MPA or PGE2/MPA. Of the seven genes, one, *IGFBP3*, significantly decreased in expression and all other genes either did not respond or increased in expression in response to *FOXO1* KD in MdESF (Fig 6C). This result suggests that FOXO1 transcriptionally regulates distinct sets of genes in MdESF and HsDSC. If we assume that the reproductive mode in MdESF is representative of the ancestral paleo-ESF, these data also suggest that the evolution of mammalian DSCs proceeded through modifying the target gene set of a largely conserved core GRN that includes *FOXO1*.

## Discussion

Here we show that, whereas human ESF respond to decidualizing stimuli with a compensated physiological phenotype, the opossum ESF exhibit a classic stress phenotype. This difference was also found, though to a lesser degree, when we used a more physiological signal, PGE2, instead of extracellular 8-br-cAMP. The responses of human and opossum ESFs were remarkably similar at the level of DSC regulatory gene expression, both in terms of transcriptional as well as post-translational regulation as in the case of FOXO1. While post-translational activation of FOXO1 is necessary in human cells for the expression of DSC effector genes, e.g., *PRL*, *IGFBP1*, etc., in opossum ESF, the functional role of activated FOXO1 is to protect the cells against apoptosis.

**Fig 7:**
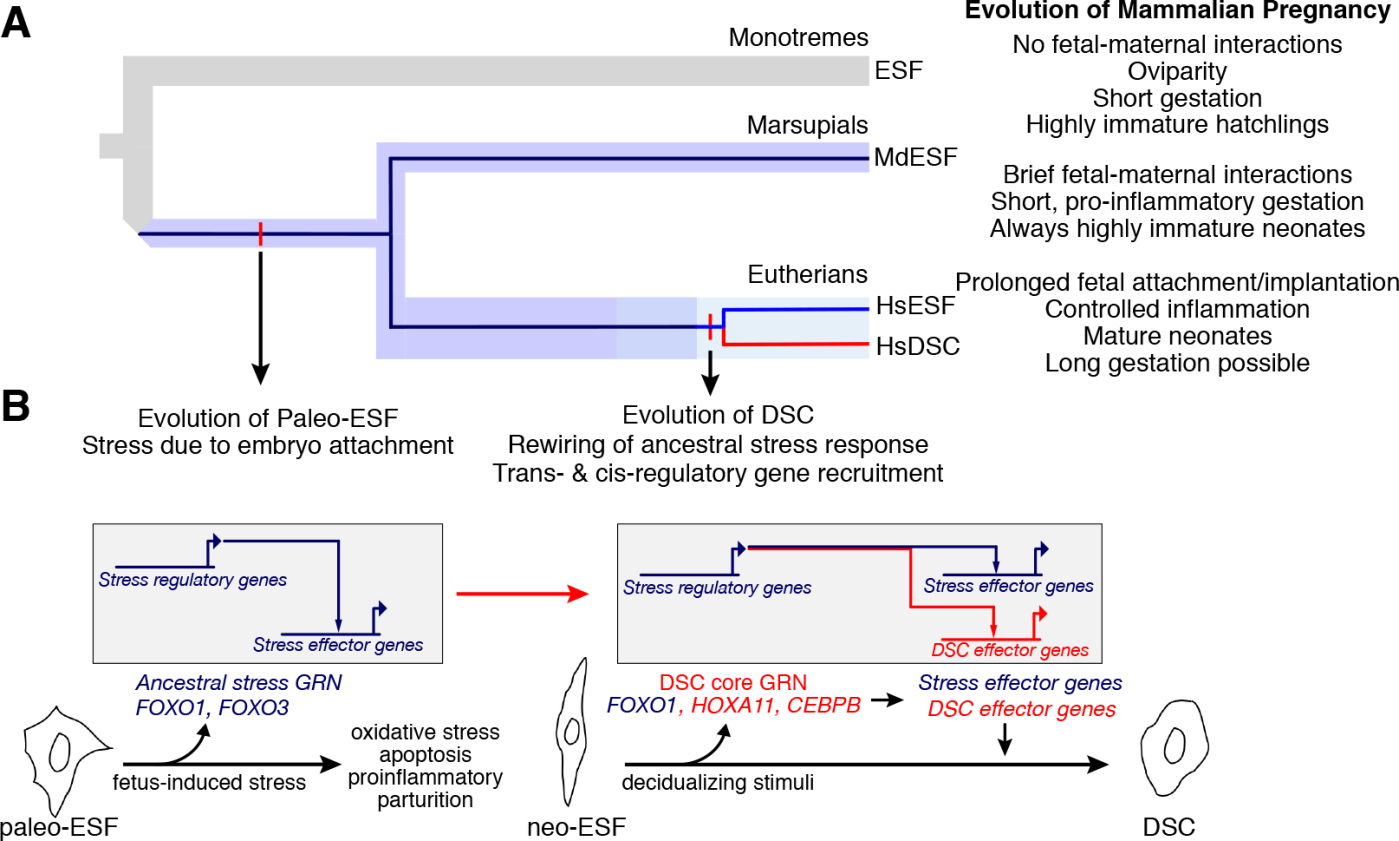
The eutherian mammal decidual cell type evolved from a cellular stress response. (A) Phylogeny showing the three major mammalian clades. Embedded in the species tree, the cell type tree for ESF and DSC cell types is drawn. Paleo-ESF is an ancestral cell type to crown group neo-ESF, such as human HsESF, which arose after the split of metatherian (marsupials) and eutherian (placental) mammals. The eutherian neo-ESF is defined by its ability to differentiate into DSC, which is the sister cell type to eutherian neo-ESF. (B) Proposed model of the evolution of eutherian DSC from a paleo-ESF ancestral cell type. Paleo-ESF underwent a stress response during the short gestational period in which fetal–maternal interactions lead the proinflammatory, stress-induced parturition seen in crown marsupials. The ancestral stem eutherian rewired the stress-related regulatory modules to stabilize an alternative gene regulatory state and control the expression of effector genes, which allow fetal implantation and invasive placentation, long gestation, and anti-inflammatory phenotypes typical of crown eutherian mammals. DSC, decidual stromal cell; ESF, endometrial stromal fibroblast; HsESF, human endometrial stromal fibroblast.

We propose that the signaling pathway and large parts of the TF network are homologous and to some degree conserved between eutherian DSC and marsupial MdESF, suggesting that these components were also present in paleo-ESF, i.e., prior to the evolutionary origin of DSCs. Furthermore, we hypothesize that there were at least two distinct molecular changes that led to the evolution of DSCs. On one hand, we find a small number of decidual TFs that are not expressed in stimulated MdESF, viz. members of the 5’ HoxD cluster *HoxD12*, *HoxD11*, and *HoxD9*, as well as *FOXM1*, *TFAP2C*, *PRRX2*, and *E2F8*. This suggests that one aspect of the evolution of the core GRN is the recruitment of additional TF genes through the evolution of cAMP response elements in existing or novel *cis*-regulatory elements. For example, in this list we find *E2F8*, which functions in regulating polyploidization [35], a derived feature of DSCs. On the other hand, we also find that DSC effector genes of the conserved TF network are different. Thus, another element by which the ancestral ESF cell type evolved into DSCs was the rewiring of gene regulation downstream of FOXO1 and other decidual regulatory genes, e.g., *CEBPB*, *PGR*, *HOXA10*, *HOXA11*, and *GATA2* (Fig 7). We tested this model by comparing the effect of *FOXO1* KD on effector gene expression and found that in fact the regulatory role of human FOXO1 in these cells is extensively different from that in opossum ESF. Exactly how this “downstream reprogramming” was effected in evolution is not known and needs to be the subject of further investigation, although previous work suggests that recruitment of transposable elements may have been a key factor [52,53].

An alternative explanation for our results is that the decidual cell type may have evolved in the stem therian lineage, i.e., before the most recent common ancestor of eutherians and marsupials, but has been lost in marsupials. Marsupial reproduction shares many plesiomorphic reproductive traits with the most basal branching mammals, the monotremes [5]. Although opossums do not lay eggs, along with the platypus, they have a relatively short gestation period and give birth to highly altricial young [54,55]. Furthermore, DSCs are necessary for maintaining pregnancy in species with the kind of invasive placentation that is only found in eutherian mammals [9]. For these reasons, it is likely that the DSC is truly a specific trait of eutherian mammals and not one with an older origin that was subsequently lost in the marsupial group.

Our model of stress-derived decidual differentiation explains a number of otherwise puzzling facts. First, there is evidence that during physiological decidualization in humans the stromal cells produce intracellular ROS that mediate the decidualization process [56–59]. Here, we show that genes responsive to decidualization stimuli in a distantly related ESF cell type are also intimately linked to stress-related decidualization in eutherians [56], e.g., *GPX3*, *SOD1*, *SOD3*, and *CAT*, and more interestingly these genes are also associated with a stress coping mechanism. Furthermore, we show that apoptosis-related genes known to play a role in decidualization, e.g., *GADD45A*, *TRAIL*, and *BCL2L11* [56,60], are also up-regulated in MdESF. In the context of these stress-related genes, we also show that FOXO1 acts as an oxidative stress sentinel that counteracts apoptosis. Moreover, decidualization is associated with ER stress [61], which we also observe through the expression of genes specifically associated with ER stress and UPR in opossum cells treated with 8-br-cAMP/MPA. Finally, a subpopulation of endometrial stromal cells undergoes cellular senescence in humans, a senescent phenotype that plays a critical role in implantation [62]. Thus, peculiar features of human decidualization, e.g., redox signaling, ER stress, and cell senescence, are readily explained by our model, wherein decidual differentiation mechanisms arose in evolution from a pregnancy-related stress response that consequently activates many of the same regulatory genes and physiological processes as are activated in decidual cells during differentiation.

Our results point to a model for the origin of a novel cell type, namely the modification of an ancestral cellular stress response. Very few studies have addressed the molecular mechanisms for the origin of novel cell types. However, two previous studies are of particular interest in regards to this. The study by Nagao and colleagues [63] investigated the leucophore pigment cell type in the perciform lineage. Experimental evidence suggests that the origin of the leucophore in the perciform lineage was achieved by a change in the functional interaction between TFs already expressed in a precursor cell type. Similarly, changes in the functional interactions of FOXD3 during the evolution of the neural crest cell type, a vertebrate novelty, is also especially noteworthy [64]. These results are broadly consistent with the model we propose for the evolution of the decidual cell type, in which similar TFs are expressed ancestrally and changes in the functional interactions between those TFs occurred that were critical for the evolution of the novel cell type. In fact, we have shown previously that, coincidental with the origin of decidual cells, the function of TF proteins, namely HOXA11 and CEBPB interacting with FOXO1, has changed [65,66]. We note these genes are among those already responsive to decidualizing signals in opossum ESF. Hence, transcription factor protein evolution may be a common feature of the origin of novel cell types.

In evolution, structural and developmental changes can result in cells being exposed to drastically altered or novel environments. In mammals, the evolution of pregnancy and in particular the evolution of extended gestation resulted in the endometrium functioning under exposure to a range of new stimuli and with new requirements for the success of reproduction. As these developmental changes occurred, it is reasonable to expect that exposure of cell types to novel tissue and developmental environments can be a source of cellular stress. These anciently conserved pathways can be a rich source of hardwired, modular components of stress GRNs. In this case, evolution can mitigate cellular stress in a variety of ways, including decreasing the stress-inducing stimulus, but also through co-option of the stress pathways into normal physiological function. Our data suggest that the decidual stromal cell type has evolved from a physiological stress response that is likely directly related to the invasion of trophoblast into maternal tissues, the condition seen in crown eutherians today.

Whereas the evidence presented here pertains to the special case of the evolution of mammalian decidual cells, the recent discovery that ROS are important physiological signaling molecules during the differentiation of other cell types, e.g., neurons [67,68] and mesenchymal cells [69] (and functioning in tumorigenesis), may indicate a role for stress responses in the evolutionary derivation of novel cell types. Some cell types with novel physiological functions could therefore be understood as fulfilling physiological needs for which the ancestral body plan cannot compensate. The evolutionary rewiring of stress responses and functional changes in the interactions of transcription factors could be a few means within a suite of mechanisms involved in the evolutionary process of cell type origination to address physiological challenges.

## Materials and methods

### Ethics statement

All animal procedures were conducted under protocols approved by the Institutional Animal Care and Use Committee (#11313) of Yale University.

### Tissue collection

Opossum uterine tissue was collected from a *M*. *domestica* colony housed at Yale University. For ESF isolation, uterine tissue was harvested from a nonpregnant *M*. *domestica* female. For immunohistochemistry and ELISA, tissue from specific stages of the reproductive cycle were collected by following a standard breeding protocol outlined in Kin and colleagues [16]. Once collected tissue was stored for immunohistochemistry analysis in 4% paraformaldehyde in PBS 24 h, then washed in 50% ethanol for 1 h, then twice in 70% ethanol for 1 h then stored in 70% ethanol at −20°C. Tissue was stored for western blot and ELISA analysis by snap freezing in liquid nitrogen and then stored at −80°C.

### Isolation of ESFs and skin fibroblasts from *M*. *domestica*

*M*. *domestica* ESFs were isolated as previously described [16]. Briefly, primary ESFs were harvested by enzymatic digestion and centrifugation combined with Percoll density gradient. The uterus of an adult female grey short-tailed opossum *M*. *domestica* was dissected, cut in half longitudinally, and cut into 2–3–mm fragments. These were digested with 0.25% trypsin-EDTA for 35 min at 37°C and digested in Dissociation Buffer (1 mg/ml collagenase, 1 mg/ml Dispase, 400 μg/ml DNaseI) for 45 min at room temperature. Cell clumps were subsequently homogenized by passage through a 22-gauge syringe. Passage through a 40-μm nylon mesh filter removed remaining fragments. This lysate was used to generate a density gradient by centrifugation at 20,000 g for 30 min. A single cell suspension was generated from this lysate and was subsequently layered onto a Percoll gradient (1.09 g/cc Percoll, GE Healthcare Life Sciences) and centrifuged at 400 g for 20 min to allow for cells to settle to their respective density layers. Using a 25-gauge needle, each 1-ml layer was removed working from low to high density and washed into 5 ml of 50-mM NaCl solution. As with previous iterations of this protocol in this laboratory, layers 6 through 8 generally contained a fairly homogeneous population of cells that outwardly exhibited fibroblast characteristics. Cells in these layers were pelleted, resuspended in growth media, and cultured in 24-well plates. To facilitate enrichment of fibroblasts versus epithelial cells, media was exchanged in each well after two hours in order to remove floating cells that had not yet attached. To validate our cell line, we conducted comparative qPCR and immunofluorescence for proteins that mark either epithelial or mesenchymal (fibroblast) cells. Transcription factors indicative of ESFs were enriched in RNA from Percoll layer 8 relative to layer 3 or RNA isolated from spleen (S1 Table). Immunofluorescence on cells from Percoll layer 8 showed enrichment of the mesenchymal protein VIMENTIN and no epithelial contamination as judged by expression of CYTOKERATIN (S1 Fig). Therefore, cells from layer 8 were used in these experiments and were propagated in a T75 culture flask by sub-passaging over a period of 2 months at 33°C. Once confluent, cells were subpassaged using Accutase (AT104, Innovative Cell Technologies) or a cell scraper. The experiments detailed here were conducted with passages 12–20. To isolate *M*. *domestica* skin fibroblasts from the animal, ethanol was applied to the skin, hair was removed, and a small sample was excised. The sample was transferred to PBS, and the subcutaneous tissue was removed by scraping the dermal side with a razor blade and forceps. The sample was cut into strips approximately 1.0 cm^2^ with a scalpel and was incubated in 0.3% trypsin-PBS for 30 min at 37°C. The epidermis was subsequently removed, and the sample was washed in ABAM-PBS with gentle shaking. The sample was transferred to a tissue culture dish and sliced into squares approximately 3 mm in size. Five to 10 of these squares were transferred to a 35-mm tissue culture dish, and a sterile 22-mm glass coverslip was placed over the samples. The samples were grown to confluency in growth media, which was refreshed every 3 days.

### Cell culture, RNAseq, and transcriptomic analyses

MdESF were cultured in growth media with no antibiotic-antimycotic, containing (per liter) the following: 15.56 g DMEM/F-12 (D2906, Sigma Aldrich), 1.2 g sodium bicarbonate, 10 ml sodium pyruvate (11360, Thermo Fisher), 1 ml ITS (354350, VWR), and 100 ml charcoal-stripped fetal bovine serum (100–119, Gemini). Media was replaced every 4 days unless otherwise stated. Over the duration of these experiments, MdESF were found to be mycoplasma free (S8 Fig), as shown by periodic PCR assays for mycoplasma contamination (30-1012K, Universal Mycoplasma Detection Kit, ATCC). For decidualizing stimuli, MdESF were cultured in growth media supplemented with cAMP-analgoue 8-bromoadenosine 3′-5′-cyclic monophosphate sodium sale (B7880, Sigma Aldrich) and progesterone-analgoue medroxyprogesterone 17-acetate (MPA) (M1629, Sigma Aldrich), at final concentrations of 0.5 mM and 1 μM, respectively. Growth media was supplemented with prostaglandin E2 (14010, Cayman Chemicals) at a final concentration of 10 μM. For RNA sequencing of unstimulated and stimulated MdESF, cells were grown to 70% confluency in T75 flasks and treated with the respective stimuli for two days prior to harvesting with Buffer RLT and subsequent processing with RNeasy Mini Kit (74104, Qiagen) following the manufacturer’s protocol. Illumina sequencing libraries were generated from RNA by Poly-A selection and sequenced by the Yale Center for Genome Analysis on the Illumina Hiseq 2500 system. For transcriptomic and GO enrichment analyses, see below under sub-heading Transcriptomic Analyses. Human ESF and DSC transcriptomic data used in these analyses were reported previously [8], and FOXO1 KD RNAseq data in HsDSC have been deposited under GEO GSE115832.

### Immunocytology

MdESF or HsESF were grown in an 8-well chamber slide (12-565-18, Fisher) to 70% confluency. After treatment, cells were fixed with 4% paraformaldehyde in PBS for 15 min at room temperature. Cells were washed 2 times in ice-cold PBS, subsequently incubated for 10 min in 0.25% Triton X-100 in PBS, and finally washed 3 times for 5 min/wash in PBS. A blocking solution was applied with 1% bovine serum albumin (BSA) and 0.25% Triton X-100 in PBS for 30 min at room temperature. Cells were then incubated in blocking solution at 4°C overnight in the following primary antibodies: 1:200 rabbit anti-cytokeratin (ab9377, Abcam); 1:200 mouse anti-vimentin (sc-6260, Santa Cruz); mouse anti-FKHR (FOXO1) (sc-374427, Santa Cruz). Cells were subsequently washed the next day 3 times for 5 min each in PBS, and secondary antibody incubation was for 1 h at room temperature in the dark. Secondary antibodies used in this study were as follows: 1:200 Alexa Fluor 555 goat anti-mouse IgG (A21422, Thermo Fisher); 1:200 Alexa Fluor 488 goat anti-rabbit IgG (A11008, Thermo Fisher). Cells were then washed 3 times for 5 min each in PBS, and nuclei were stained with DAPI (10236276001, Roche). Finally, cells were washed one time for 5 min in PBS and observed with an Eclipse E600 microscope (Nikon) equipped with a Spot Insight camera. Lastly, it should be noted that we tested two different antibodies targeting human FOXO3 in *M*. *domestica*. These antibodies were anti-FOXO3a/FKHRL1 (EMD Millipore 07–702) and anti-FKHRL1 D-12 (Santa Cruz, Sc-48348). Both of these antibodies produced nonspecific bands on western blot and pervasive signal in MdESF. Therefore, we were not able to assess the protein localization dynamics of FOXO3 in MdESF.

### Western blot analysis

MdESF or HsESF were cultured in T75 flasks to 80% confluency. Cells were rounded up with Tryple and homogenized in RIPA buffer (89900, Thermo Fisher) supplemented with HALT Protease Inhibitor Cocktail (PI87785, Thermo Fisher) for 15 min. Suspensions were centrifuged for 15 min at 13,000 RPM at 4°C. Protein concentrations were determined with Pierce BCA Protein Assay Kit (23225, Thermo Fisher). Cell lysates were diluted to achieve a solution with 30–60 μg total protein, combined with an equal volume of 2× NuPage LDS Sample Buffer (NP007, Thermo Fisher) with 2× NuPage Sample Reducing Agent (NP0004, Thermo Fisher), heated to 70°C, loaded in a NuPage 4%–12% Bis-Tris gel (NP0321BOX, Thermo Fisher), and electrophoresed at 130 volts for 60–90 min. Proteins were transferred to polyvinylidene difluoride membranes with the iBlot Gel Transfer System (Thermo Fisher). Membranes were subsequently incubated for 1 h at room temperature in blocking buffer (3% BSA in PBST) and incubated with primary antibodies, listed above, overnight at 4°C. Primary antibody dilutions were the following: 1:200 FOXO1, VIMENTIN, and CYTOKERATIN. After primary incubation, membranes were then washed 3 times for 5 min each in PBST and subsequently incubated with the corresponding HRP-conjugated secondary antibody, 1:5,000 of either goat anti-mouse (sc-2005, Santa Cruz) or goat anti-rabbit (sc-2054, Santa Cruz). Signal was detected by incubating membranes in Clarity Western ECL substrate (1705060, Bio-Rad) in the dark for 5 min and visualized with a Bio-Rad Gel Doc System. Uncropped western blots are provided in S9 Fig and S10 Fig.

### Immunohistochemistry

Uterine tissue was dehydrated through a graded ethanol series, cleared in toluene, and then embedded in paraffin. We cut 7-μm cross sections on a microtome and mounted on Shandon polysine precleaned microscope slides (6776215Cs, Thermo Fisher). Sections were stored in the dark at room temperature until they were stained. We localized the expression of FOXO1 using immunohistochemistry. Slides were deparafinized in three successive washes of xylene (3 min each), then three successive washes of ethanol (3 min). Antigen retrieval was performed in citrate buffer (12 mM sodium citrate, pH 6.0, 98°C, 1 h). Endogenous peroxidase activity was blocked with Dako Peroxidase Block (Dako, 30 min). Slides were then incubated in primary antibody overnight. We used a goat polyclonal antibody generated against the N-terminal of human FOXO1 protein (1:5,000 dilution, FKHR antibody sc-9809, SantaCruz). On day two, slides were incubated in a donkey anti-goat IgG-HRP secondary (1 h, 1:200 dilution, sc-2056 SantaCruz). Slides were then rinsed in PBS (5 min), PBS-BSA (0.1%, 5 min), then incubated for 5 minutes in TSA Plus Cyanine 3 system (1:50 NEL744001KT, PerkinElmer Inc.). Slides were again washed in PBS (5 min) and PBS-BSA (0.1%, 5 min), counter stained in DAPI (1 time, 2 min, 10236276001; Roche), washed in deionized water for 5 min, and mounted in glycerol (50%).

### Prostaglandin E2 ELISA

Snap frozen uterine tissue from adult female *M*. *domestica* was homogenized in extraction buffer (0.1 M phosphate, 1 mM EDTA, pH 7.4) containing indomethacin (10 μM final concentration) using a mechanical homogenizer (TissueRuptor, QIAGEN). Cellular debris was removed by centrifugation (>13,000 RPM, 4 ^o^C, 10 min). Tissue lysates were aliquoted into single use tubes and frozen at −80°C. Protein concentration was measured using Pierce BCA Protein Assay Kit (23225, Thermo Fisher). After determining protein concentration, 1 mg of protein for each sample was used in the first round of ELISA against PGE2 following the manufacturer’s protocol (514010, Cayman Chemical). Each sample was run in duplicate at two different dilutions. A dilution series of PGE2 standard provided by the manufacturer was included in each run, and PGE2 values in ng per mg protein were calculated from these standards. Due to the sensitivity of this assay, some samples contained excess PGE2 and therefore required additional dilutions in order to fall within the calculable range of the standard. For these samples with excess PGE2, an additional ELISA plate was run with two additional dilutions. Samples were incubated in the provided PGE2 monoclonal antibody ELISA plate overnight at 4°C. The following day, the wells were washed 3 times and the staining reaction was allowed to proceed for 45 min with shaking at 400 RPM on an orbital shaker in the dark. The plate was read at 405 nm on a Viktor X multilight plate reader (Perkin Elmer).

### Quantitative PCR

MdESF were cultured in T25 culture flasks to 70% confluency and subsequently transfected with siRNAs as above. After two days, cells were treated either with 8-br-cAMP/MPA or with PGE2/MPA for four days. The change of media was accompanied by an additional round of siRNA transfection per above. At the time of RNA harvest, media was removed, cells were washed 2 times in PBS, and cells were lysed directly in the flask with Buffer RLT Plus plus beta-mercaptoethanol. RNA was harvested according to the manufacturer’s protocol (74034, RNeasy Plus Micro Kit, Qiagen). Reverse transcription of 3 μg of RNA was carried out with iScript cDNA Synthesis Kit (1708891, Bio-Rad) with an extended transcription step of three hours at 42°C. For qPCR, all reactions were carried out with Power SYBR Green PCR Master Mix (4368708, Thermo Fisher) in triplicate with 40 ng of cDNA for template in each technical replicate reaction. Fold change was calculated by finding the ddCt values relative to the expression of TATA Binding Protein. All qPCR primer sets were validated by analysis of melting curves for 2 different sets of primers for the same gene. Primer sets used in this study are listed in S2 Table.

### RNA interference

MdESF at 70% confluency in 6-well culture plates were transfected with siRNAs targeting *FOXO1* (Mission custom siRNA, V30002, Sigma Aldrich), *FOXO3* (Mission custom siRNA, V30002, Sigma Aldrich), or negative control scrambled siRNA (Silencer Negative Control No. 1, AM4611, Thermo Fisher). In preparation for transfection, siRNAs in 37.5 μl of OptiMem I Reduced Serum Media (31985, Thermo Fisher) were mixed with an equal volume of OptiMem containing 1.5 μl of Lipofectamine RNAiMax (13778, Thermo Fisher), incubated at room temperature for 15 min, and subsequently added dropwise to cells in 3 ml growth media. Final concentration of siRNAs was 25 nM. In experiments involving stimulated media, an additional round of siRNA transfection was prepared and added dropwise after growth media with decidualizing stimuli was added. Two siRNAs were transfected for each gene. Custom siRNAs were synthesized by Sigma to target the mRNAs of *M*. *domestica FOXO1* and *FOXO3*, using the NCBI Reference Sequences XM_001368275.4 (*FOXO1*) and XM_001368456.2 (*FOXO3*). Sense and antisense sequences are listed in S3 Table. siRNA-mediated KD of human *FOXO1* in human decidual cells was carried out as previously described [70].

We confirmed depletion of both RNA and protein by qPCR and western blot. For FOXO1, qPCR analyses showed that KD efficiency for these pooled siRNAs was >90% (S7B Fig). This depletion in RNA led to a corresponding depletion in FOXO1 protein, as confirmed by western blot (S10 Fig). We also desired to conduct a protein-level analysis for *M*. *domestica* FOXO3. However, for *M*. *domestica* FOXO3, we were able to confirm RNA depletion only (S7B Fig), as we did not find a commercially available antibody that showed high specificity for FOXO3 in *M*. *domestica*.

### Flow cytometry including ROS and apoptosis detection

Cells were transfected as above with RNAi reagents and subsequently incubated in growth media in the presence of decidualizing stimuli for four days. For FACS analyses, conditioned media from each well was decanted into separate 15-ml conical centrifuge tubes, and cells were then washed in 2 ml PBS. PBS was decanted into the same 15-ml conical tube as the conditioned media, and 250 μl warm Tryple Express (12604, Thermo Fisher) was added to each well. To facilitate detachment, cells were placed in a 33°C incubator for 10 min. The detachment reaction was stopped by adding 700 μl growth media to each well. Cells were then transferred to their separate 15-ml conical tubes with conditioned media and PBS and subsequently pelleted by centrifugation at 400 RPM for 5 min. Supernatant was removed, and each cell pellet was resuspended in 1 ml pre-warmed Hank’s Balanced Salt Solution (HBSS) without Phenol Red, Ca^2+^, Mg^2+^ (10–547, Lonza) containing freshly resuspended H2DCFDA (Image-IT LIVE Green ROS Detection Kit, I36007, Thermo Fisher) to a final concentration of 25 μM. Cells were incubated at 33°C for 40 min. Just prior to FACS analysis, cells were placed on ice, and 0.5 ml HBSS containing 1 μg/ml propidium iodide was added to each tube. We utilized a doublet discrimination gating strategy on a BD Aria FACS instrument, wherein on average 93% of all cells were included in the analyses (S11 Fig). Fluorescent signal detected in scrambled siRNA negative control cells were utilized to set the quadrant boundaries. Data for three replicates of each experiment were collected and mean percentages for each quadrant were calculated.

### Transcriptomic analyses

Raw sequencing reads were mapped to opossum *M*. *domestica* genome assembly monDom5 with Ensembl annotation v86, using Tophat2 v2.1.1 [71] and Bowtie2 v2.2.9 [72]. Read counts for all genes were calculated using HTSeq v0.6.1p1 [73] with Python (v2.7.13). Transcripts per million were calculated to estimate relative mRNA abundance [74]. We used Bioconductor package edgeR v3.16.5 [75] to assay for differential gene expression between unstimulated and stimulated MdESF. Genes that met the following criteria were considered to be significantly differentially expressed: (1) change in expression of at least 1.5-fold; (2) resulted in an adjusted *P*-value smaller than 10^−6^; and (3) expressed in at least one condition under comparison (TPM ≥ 3) [76]. GO enrichment analyses were performed using Gorilla [77], and the results were visualized using REViGO [78].

### Statistical analyses

The data from the experiment testing the effect of 8-br-cAMP/MPA and PGE2/MPA, as well as FOXO1 and FOXO3 KDs on the presence of ROS and apoptotic cells, were analyzed as a two-factor ANOVA. The two factors were “stimulation” and “treatment,” where “stimulation” had the levels, “growth media,” 8-br-cAMP/MPA, and PGE2/MPA and “treatment” had the levels random siRNA, FOXO1 KD, and FOXO3 KD. The response variable was the fraction of cells showing either ROS or PI fluorescence. The analysis was performed with raw frequencies as well as with log-transformed response variables. For the PGE2 ELISA, the concentration of PGE2 for each sample was determined by standard curve. The values were subsequently log transformed and used in a one-tailed *t* test.

## Supporting information

S1 Fig Immunofluorescence of VIMENTIN and CYTOKERATIN in unstimulated MdESF. (A-B) Two different passages after isolation from nonpregnant *M*. *domestica* are shown.
(TIF)

S2 Fig Analysis of decidualization regulatory genes in MdESF and *M. domestica* skin fibroblasts (MdSF) treated with 8-br-cAMP/MPA or PGE2/MPA or siRNA targeting GR. (A) Expression of 28 decidualization transcription factor genes in MdESF in response to 2-day treatment with either 8-br-cAMP/MPA (black bars) or PGE2/MPA (grey bars). Nonparametric correlation of expression between treatments and across genes was found to be 0.863 (** Spearman’s rho, *p* = 3.46 × 10^−9^). Error bars are standard error of the mean. (B) RNA abundance of MPA-responsive regulatory genes in MdESF treated with MPA for 2 days and siRNA targeting GR mRNA. Red, dashed bars show 2-fold up- or down-regulation relative to control treated with MPA alone for 2 days and negative control siRNA. Error bars represent the standard deviation of two replicates. (C) RNA abundance of select decidualization regulatory genes in response to 2-day treatment with either 8-br-cAMP/MPA or PGE2/MPA relative to control in skin fibroblasts isolated from *M*. *domestica*. Red, dashed bars show 2-fold up- or down-regulation relative to control treated with growth media alone. (D) Visualization of GO term clusters associated with 2-day treatment of 8-br-cAMP/MPA treatment in MdESF. Colored boxes represent semantic similiarity. Size of boxes represents *P*-value assigned to that cluster. cAMP, cyclic AMP; GR, glucocorticoid receptor; MPA, medroxyprogesterone acetate; PGE2, prostaglandin E2; siRNA, small interfering RNA
(TIF)

S3 Fig Effect of 8-br-cAMP concentration and removal of decidualizing stimuli on morphology of MdESF. (A) Concentration-dependent morphological response of MdESF to 8-br-cAMP/MPA treatment after 6 hours (top panels) and 22 hours (bottom panels). (B) MdESF morphological response to 8-br-cAMP/MPA treatment is reversible at higher 8-br-cAMP concentrations after 1 day in growth media. Images were taken two hours after treatment, at which time media with decidualizing stimuli were changed to growth media. Images were subsequently acquired after 19 hours in growth media. Scale bars are 10 μm. (C) Differential interference contrast images showing morphological response of MdESF treated with either MPA or 8-br-cAMP/MPA for 2 days. Images obtained during ROS detection (see Fig 1D). cAMP, cyclic AMP; MPA, medroxyprogesterone acetate; ROS, reactive oxygen species
(TIF)

S4 Fig Transcriptional response of KEGG pathway genes associated with prostaglandin signaling in MdESF and ROS detection in MdESF treated with PGE2/MPA. (A) Morphological response of MdESF treated with PGE2 alone for 2 days (B) Flow cytometry contour plots showing ROS (H2DCFDA) versus staining for apoptosis (propidium iodide). Quadrants are set to maximum extent of boundaries in unstimulated MdESF. Q1, ROS positive only; Q2, ROS and apoptosis positive; Q3, negative for ROS and apoptosis; Q4, negative for ROS and positive for apoptosis. Percentage in each quadrant for this replicate is shown. Note increase of ROS positive cells after PGE2/MPA 3-day treatment, similar to what was found with 8-br-cAMP/MPA (*p* = 0.0186). (C) Decidualization core regulatory genes do not respond in MdESF when treated with PGE2 alone for 2 days. Blue dots represent significant differential expression relative to unstimulated MdESF (*n* = 3, *p* < 10^−6^). Grey dots represent no significant change in expression. Each point represents the mean of three replicates. H2DCFDA, 2′,7′ dichlorodihydrofluorescein diacetate; KEGG, Kyoto Encyclopedia of Genes and Genomes; MPA, medroxyprogesterone acetate; PGE2, prostaglandin E2; ROS, reactive oxygen species
(TIF)

S5 Fig GO clusters from differentially expressed up-regulated and down-regulated genes for the 2-day PGE2/MPA treatment group as visualized by REViGO treemaps. Treemaps are unedited and were produced using the R script available at REViGO. Color of the boxes represents semantic similarity. Size of the boxes represents *P*-value of each cluster. (A) Differentially expressed up-regulated genes in PGE2/MPA. (B) Differentially expressed down-regulated genes in PGE2/MPA. GO, gene ontology; MPA, medroxyprogesterone acetate; PGE2, prostaglandin E2
(TIF)

S6 Fig GO clusters from differentially expressed up-regulated and down-regulated genes for the 2-day PGE2 treatment group as visualized by REViGO treemaps. Treemaps are unedited and were produced using the R script available at REViGO. Color of the boxes represents semantic similarity. Size of the boxes represents *P*-value of each cluster. (A) Differentially expressed up-regulated genes in PGE2. (B) Differentially expressed down-regulated in PGE2. GO, gene ontology; PGE2, prostaglandin E2
(TIF)

S7 Fig Immunofluorescence of FOXO1 in HsESF and FOXO1/FOXO3 KD in MdESF. (A) Although *FOXO1* RNA is present in HsESF, FOXO1 protein is constantly marked for degradation by AKT dependent polyubiquitination. In the presence of MPA for 2 days, degradation of FOXO1 protein is disrupted, and FOXO1 disproportionately loads in the cytoplasm relative to the nucleus, though some cells are positive for nuclear FOXO1. In the presence of 8-br-cAMP/MPA for 2 days, FOXO1 protein loads disproportionately in the nucleus relative to the cytoplasm in HsESF. Scale bars are 20 μm. (B) Fold change of *FOXO1* and *FOXO3* RNA in cells treated for 2 days with siRNA targeting *FOXO1* and *FOXO3* relative to scrambled siRNA control. siRNAs targeting *FOXO1* and *FOXO3* RNA removed greater than 90% of *FOXO1* and *FOXO3* transcripts. (C) Western blot for FOXO1 in total protein lysates collected from MdESF treated with 8-br-cAMP/MPA for 3 days or 5 days and with siRNA targeting *FOXO1* RNA. AKT, protein kinase B; cAMP, cyclic AMP; FOXO, forkhead box class O; KD, knockdown; MPA, medroxyprogesterone acetate
(TIF)

S8 Fig (A-F) Gel images of PCR amplification for mycoplasma contamination.
(TIF)

S9 Fig (A-C) Uncropped images of western blots for antibodies in this study.
(TIF)

S10 Fig (A) Uncropped western blot of FOXO1 protein in MdESF in presence of 8-br-cAMP/MPA for 3 and 5 days and FOXO1-specific siRNAs, as well as FOXO1 presence in total protein extracts from pregnant *M*. *domestica* uterus. FOXO, forkhead box class O; MPA, medroxyprogesterone acetate
(TIF)

S11 Fig Gating for flow cytometry analysis. (TIF)

S1 Table Assessment by qPCR of ESF markers on RNA isolated from *M. domestica* spleen tissue and on RNA isolated from two different layers in the Percoll density gradient on *M. domestica* uterine tissue. Values shown are fold enrichment relative to TATA Binding Protein (TBP) in each sample. ESF, endometrial stromal fibroblast
(XLSX)

S2 Table qPCR primers used in this study. (DOCX)

S3 Table Sequences for siRNAs used in this study. siRNA, small interfering RNA
(DOCX)

S1 Movie Time lapse micrographs of morphological response of *M. domestica* ESFs upon exposure to 8-br-cAMP/MPA. Over the first hour of treatment, micrographs were taken every 30 seconds and subsequently spliced together. cAMP, cyclic AMP; ESF, endometrial stromal fibroblast; MPA, medroxyprogesterone acetate
(MOV)

S1 Data (XLSX)

S2 Data (XLSX)

## Abbreviations

Akt: protein kinase B
BSA: bovine serum albumin
cAMP: cyclic AMP
d.p.c.: days post coitus
DSC: decidual stromal cell
ER: endoplasmic reticulum
ESF: endometrial stromal fibroblast
FOXO: forkhead box class O
GO: gene ontology
GR: glucocorticoid receptor
GRN: gene regulatory network
H2DCFDA: 2′,7′ dichlorodihydrofluorescein diacetate
HBSS: Hank’s Balanced Salt Solution
HsDSC: human decidual stromal cell
HsESF: human endometrial stromal fibroblast
KD: knockdown
KEGG: Kyoto Encyclopedia of Genes and Genomes
MdESF: opossum *M*. *domestica* endometrial stromal fibroblast
MdSF: opossum *M*. *domestica* skin fibroblasts
MPA: medroxyprogesterone acetate
PGE2: prostaglandin E2
PI: propidium iodide
PKA: protein kinase A
PTGES: prostaglandin E synthase
PTGS: prostaglandin synthase
RLN: relaxin
RNAseq: RNA sequencing
ROS: reactive oxygen species
siRNA: small interfering RNA
TBHP: tert-butyl hydrogen peroxide
TF: transcription factor
TPM: transcripts per million
TRAIL: TNF-related apoptosis-inducing ligand
UPR: unfolded protein response
