# Supplementary figures and images for: The mammalian decidual cell evolved from a cellular stress response
Source: PLoS Biol. 2018 Aug 24;16(8):e2005594. doi: 10.1371/journal.pbio.2005594 (PMC6108454; doi:10.1371/journal.pbio.2005594)

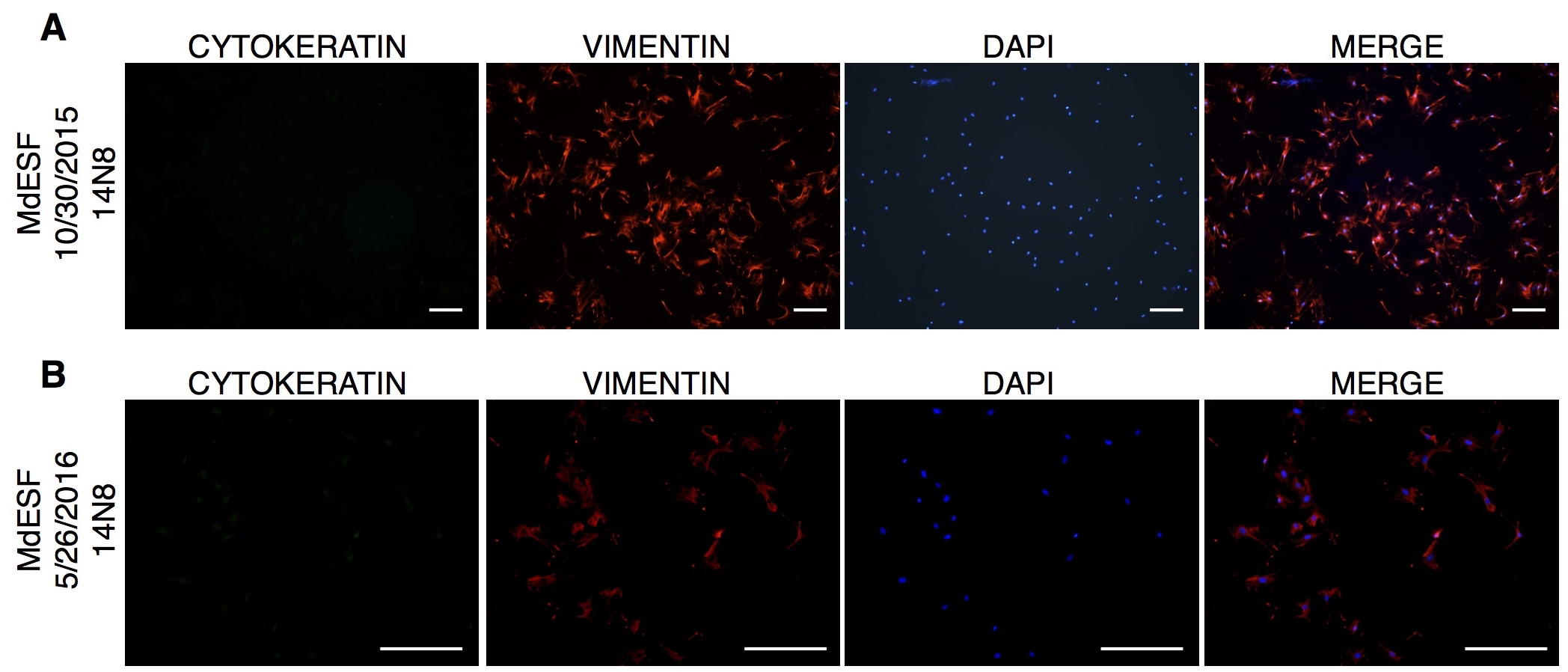

Supplement: S1 Fig — (A-B) Two different passages after isolation from nonpregnant M. domestica are shown. (TIF) [file pbio.2005594.s001.tif]

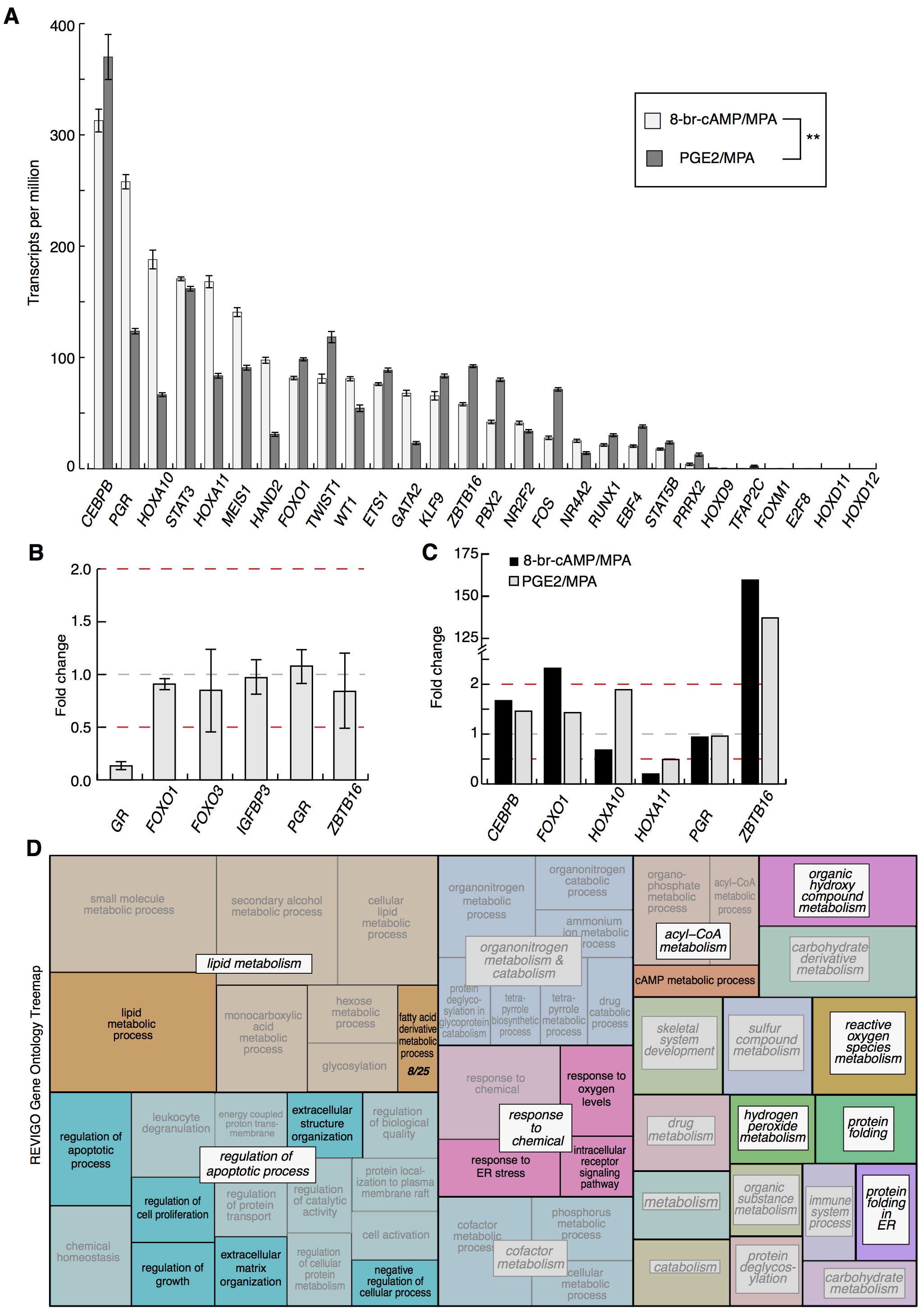

Supplement: S2 Fig — (A) Expression of 28 decidualization transcription factor genes in MdESF in response to 2-day treatment with either 8-br-cAMP/MPA (black bars) or PGE2/MPA (grey bars). Nonparametric correlation of expression between treatments and across genes was found to be 0.863 (** Spearman’s rho, p = 3.46 × 10^−9^). Error bars are standard error of the mean. (B) RNA abundance of MPA-responsive regulatory genes in MdESF treated with MPA for 2 days and siRNA targeting GR mRNA. Red, dashed bars show 2-fold up- or down-regulation relative to control treated with MPA alone for 2 days and negative control siRNA. Error bars represent the standard deviation of two replicates. (C) RNA abundance of select decidualization regulatory genes in response to 2-day treatment with either 8-br-cAMP/MPA or PGE2/MPA relative to control in skin fibroblasts isolated from M. domestica. Red, dashed bars show 2-fold up- or down-regulation relative to control treated with growth media alone. (D) Visualization of GO term clusters associated with 2-day treatment of 8-br-cAMP/MPA treatment in MdESF. Colored boxes represent semantic similiarity. Size of boxes represents P-value assigned to that cluster. cAMP, cyclic AMP; GR, glucocorticoid receptor; MPA, medroxyprogesterone acetate; PGE2, prostaglandin E2; siRNA, small interfering RNA (TIF) [file pbio.2005594.s002.tif]

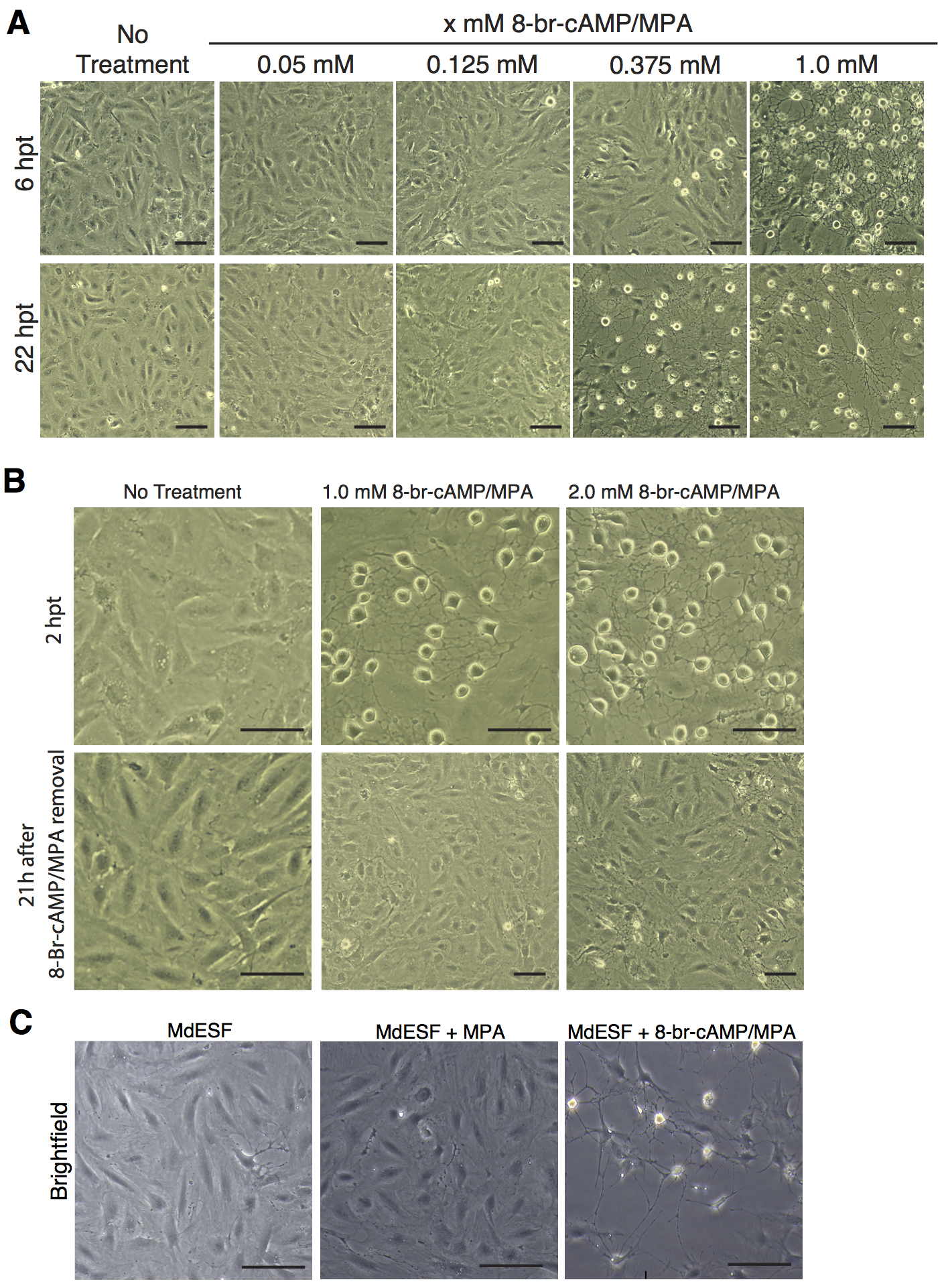

Supplement: S3 Fig — (A) Concentration-dependent morphological response of MdESF to 8-br-cAMP/MPA treatment after 6 hours (top panels) and 22 hours (bottom panels). (B) MdESF morphological response to 8-br-cAMP/MPA treatment is reversible at higher 8-br-cAMP concentrations after 1 day in growth media. Images were taken two hours after treatment, at which time media with decidualizing stimuli were changed to growth media. Images were subsequently acquired after 19 hours in growth media. Scale bars are 10 μm. (C) Differential interference contrast images showing morphological response of MdESF treated with either MPA or 8-br-cAMP/MPA for 2 days. Images obtained during ROS detection (see Fig 1D). cAMP, cyclic AMP; MPA, medroxyprogesterone acetate; ROS, reactive oxygen species (TIF) [file pbio.2005594.s003.tif]

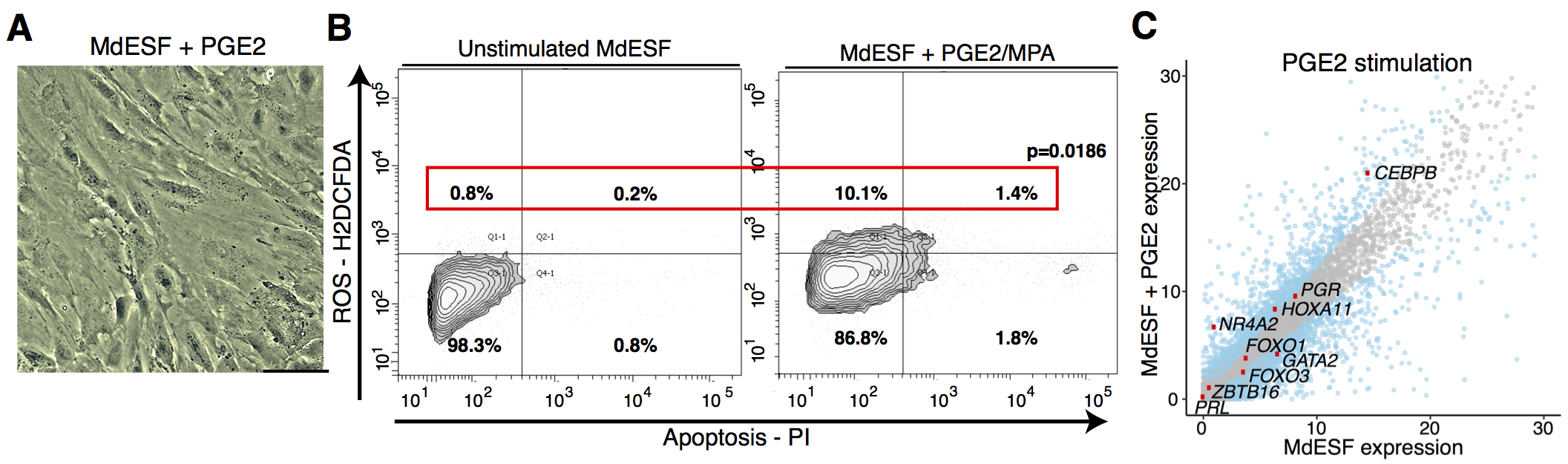

Supplement: S4 Fig — (A) Morphological response of MdESF treated with PGE2 alone for 2 days (B) Flow cytometry contour plots showing ROS (H2DCFDA) versus staining for apoptosis (propidium iodide). Quadrants are set to maximum extent of boundaries in unstimulated MdESF. Q1, ROS positive only; Q2, ROS and apoptosis positive; Q3, negative for ROS and apoptosis; Q4, negative for ROS and positive for apoptosis. Percentage in each quadrant for this replicate is shown. Note increase of ROS positive cells after PGE2/MPA 3-day treatment, similar to what was found with 8-br-cAMP/MPA (p = 0.0186). (C) Decidualization core regulatory genes do not respond in MdESF when treated with PGE2 alone for 2 days. Blue dots represent significant differential expression relative to unstimulated MdESF (n = 3, p < 10−6). Grey dots represent no significant change in expression. Each point represents the mean of three replicates. H2DCFDA, 2′,7′ dichlorodihydrofluorescein diacetate; KEGG, Kyoto Encyclopedia of Genes and Genomes; MPA, medroxyprogesterone acetate; PGE2, prostaglandin E2; ROS, reactive oxygen species (TIF) [file pbio.2005594.s004.tif]

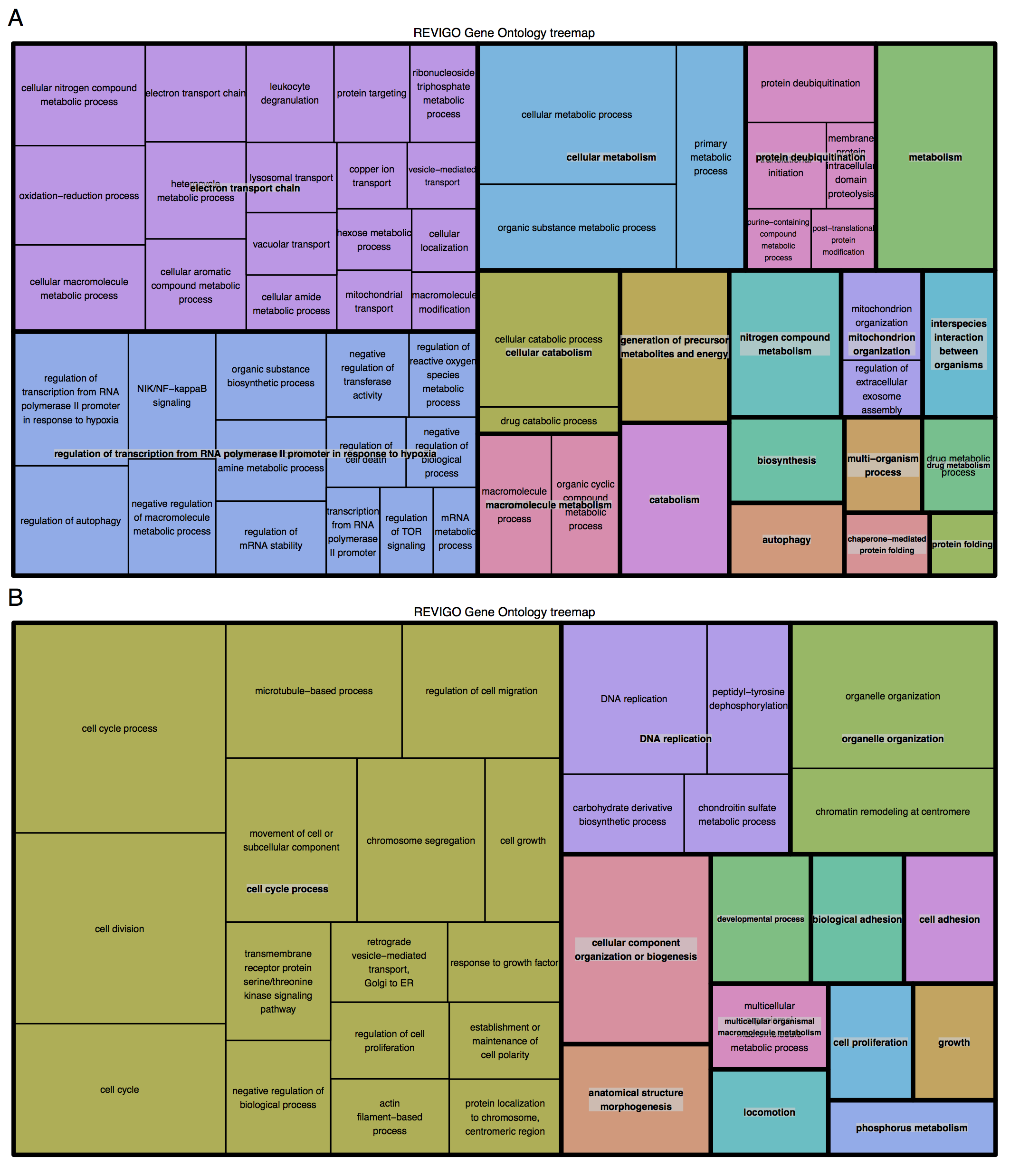

Supplement: S5 Fig — Treemaps are unedited and were produced using the R script available at REViGO. Color of the boxes represents semantic similarity. Size of the boxes represents P-value of each cluster. (A) Differentially expressed up-regulated genes in PGE2/MPA. (B) Differentially expressed down-regulated genes in PGE2/MPA. GO, gene ontology; MPA, medroxyprogesterone acetate; PGE2, prostaglandin E2 (TIF) [file pbio.2005594.s005.tif]

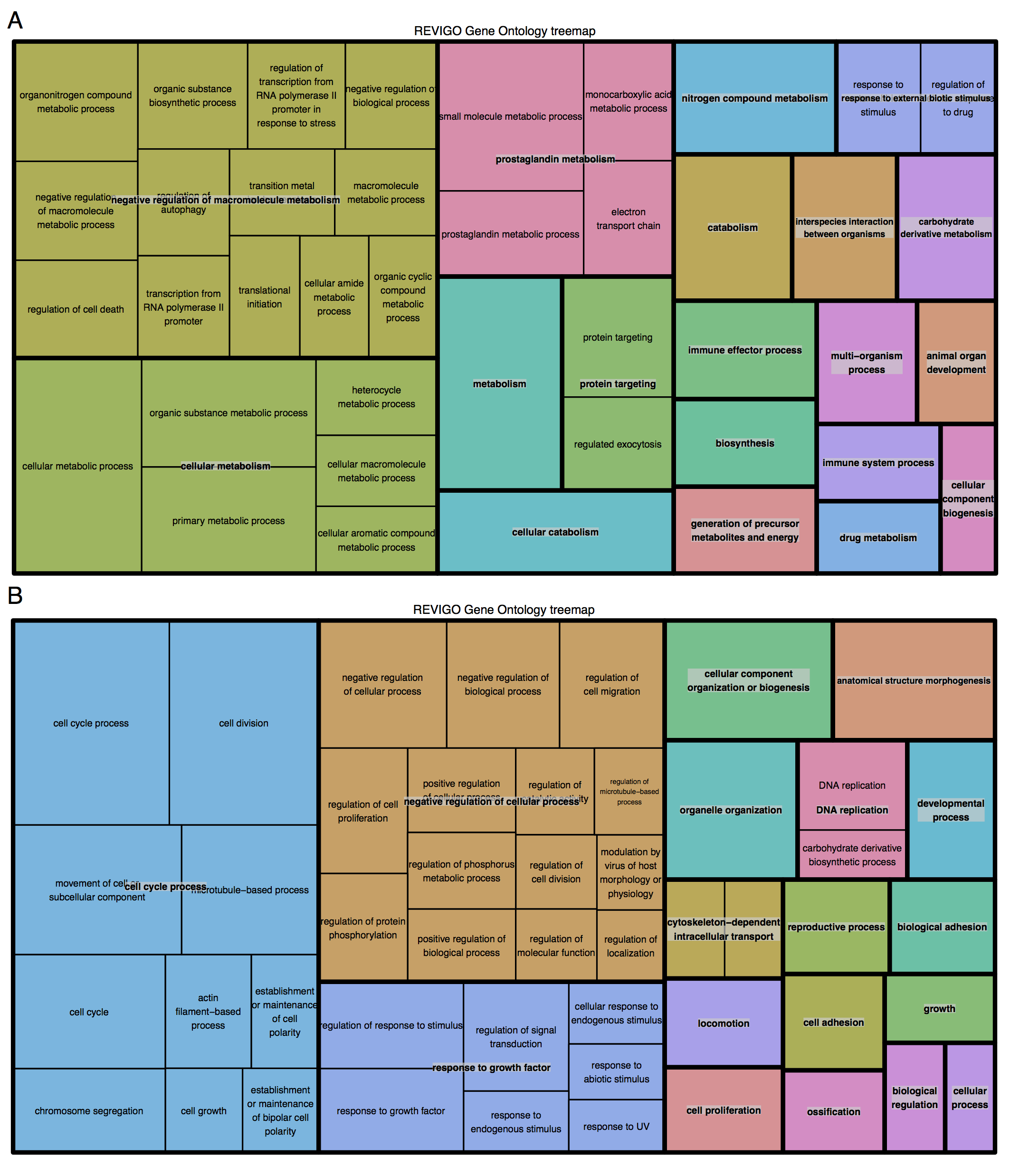

Supplement: S6 Fig — Treemaps are unedited and were produced using the R script available at REViGO. Color of the boxes represents semantic similarity. Size of the boxes represents P-value of each cluster. (A) Differentially expressed up-regulated genes in PGE2. (B) Differentially expressed down-regulated in PGE2. GO, gene ontology; PGE2, prostaglandin E2 (TIF) [file pbio.2005594.s006.tif]

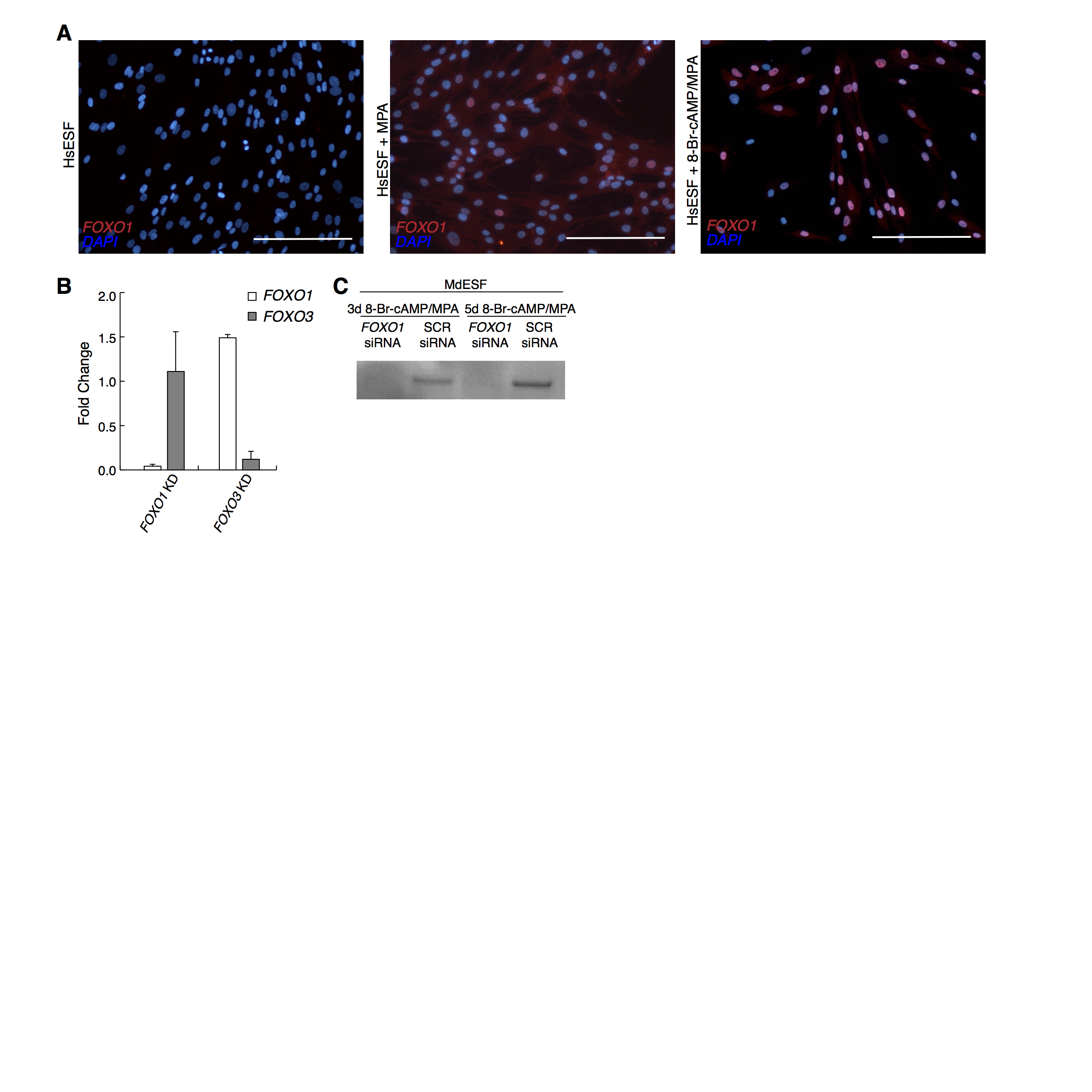

Supplement: S7 Fig — (A) Although FOXO1 RNA is present in HsESF, FOXO1 protein is constantly marked for degradation by AKT dependent polyubiquitination. In the presence of MPA for 2 days, degradation of FOXO1 protein is disrupted, and FOXO1 disproportionately loads in the cytoplasm relative to the nucleus, though some cells are positive for nuclear FOXO1. In the presence of 8-br-cAMP/MPA for 2 days, FOXO1 protein loads disproportionately in the nucleus relative to the cytoplasm in HsESF. Scale bars are 20 μm. (B) Fold change of FOXO1 and FOXO3 RNA in cells treated for 2 days with siRNA targeting FOXO1 and FOXO3 relative to scrambled siRNA control. siRNAs targeting FOXO1 and FOXO3 RNA removed greater than 90% of FOXO1 and FOXO3 transcripts. (C) Western blot for FOXO1 in total protein lysates collected from MdESF treated with 8-br-cAMP/MPA for 3 days or 5 days and with siRNA targeting FOXO1 RNA. AKT, protein kinase B; cAMP, cyclic AMP; FOXO, forkhead box class O; KD, knockdown; MPA, medroxyprogesterone acetate (TIF) [file pbio.2005594.s007.tif]

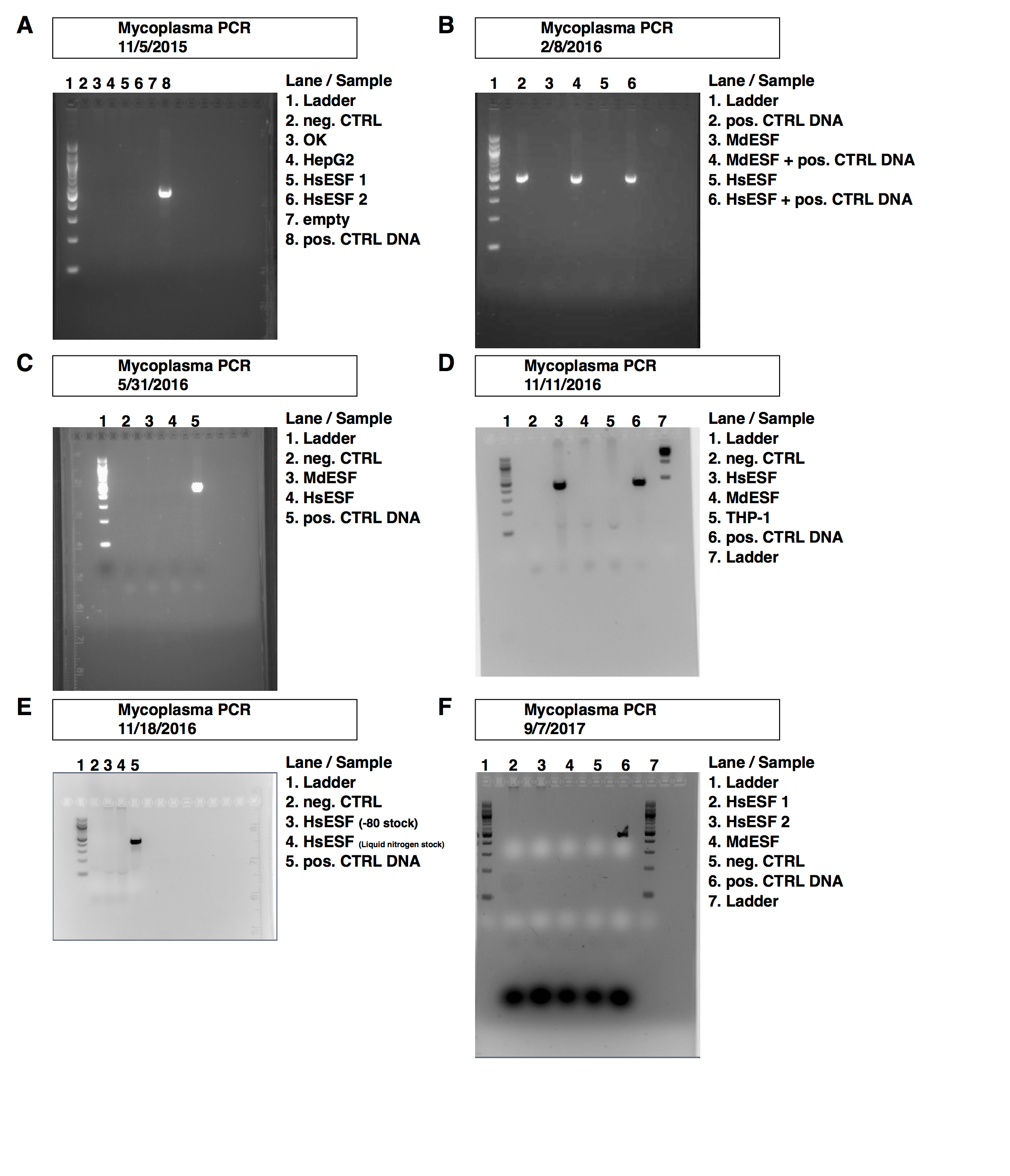

Supplement: S8 Fig — (A-F) Gel images of PCR amplification for mycoplasma contamination. (TIF) [file pbio.2005594.s008.tif]

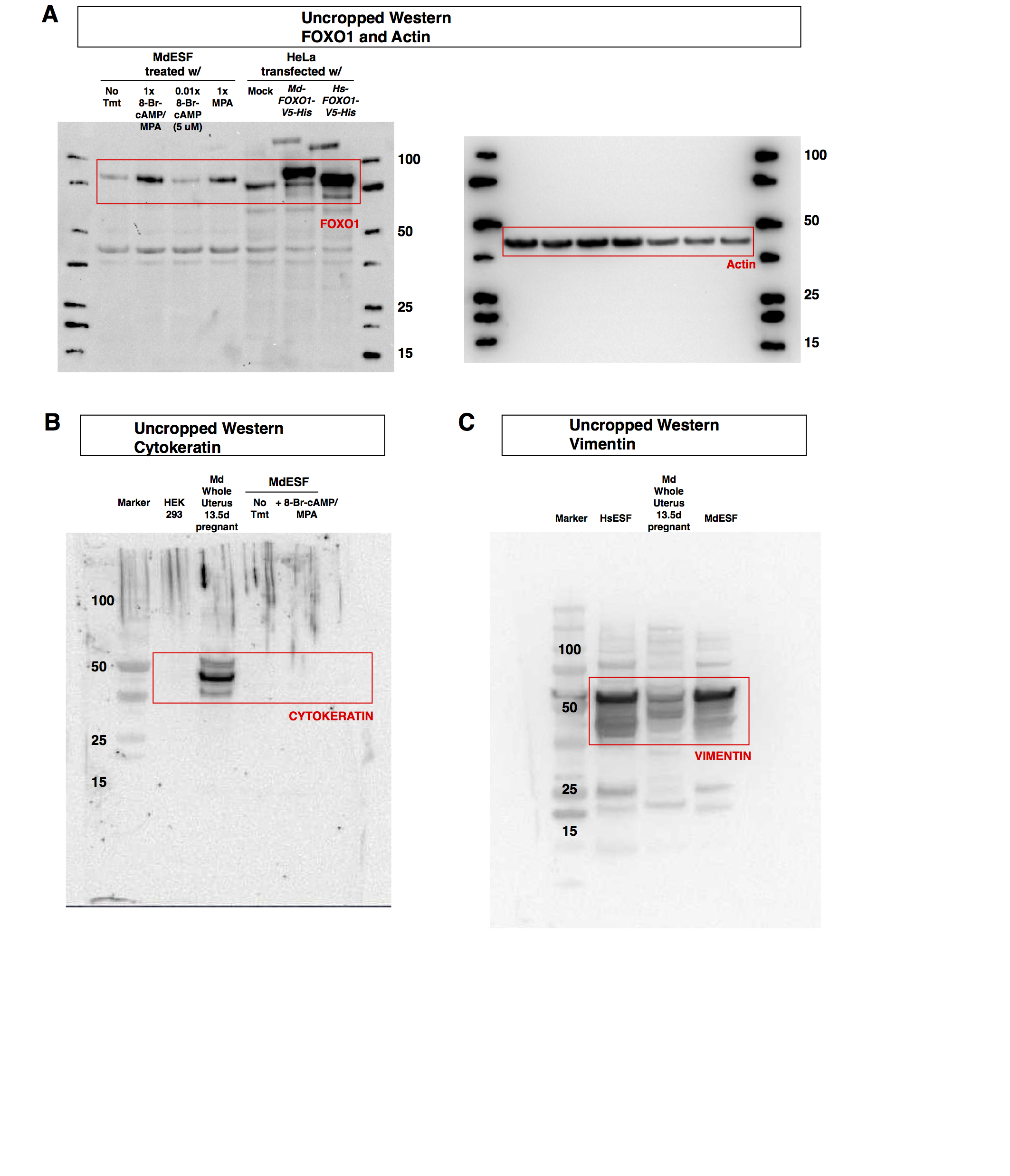

Supplement: S9 Fig — (A-C) Uncropped images of western blots for antibodies in this study. (TIF) [file pbio.2005594.s009.tif]

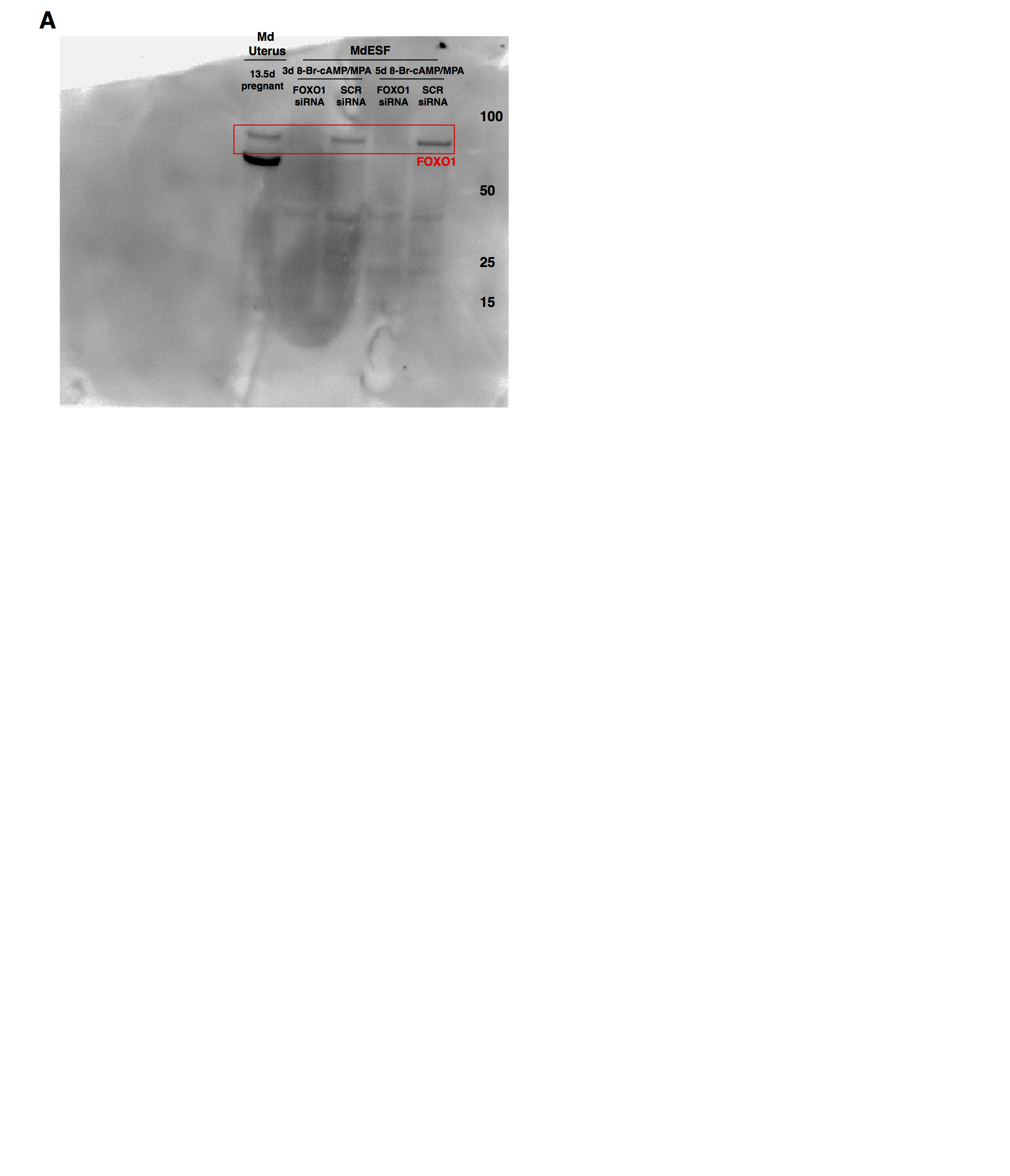

Supplement: S10 Fig — (A) Uncropped western blot of FOXO1 protein in MdESF in presence of 8-br-cAMP/MPA for 3 and 5 days and FOXO1-specific siRNAs, as well as FOXO1 presence in total protein extracts from pregnant M. domestica uterus. FOXO, forkhead box class O; MPA, medroxyprogesterone acetate (TIF) [file pbio.2005594.s010.tif]

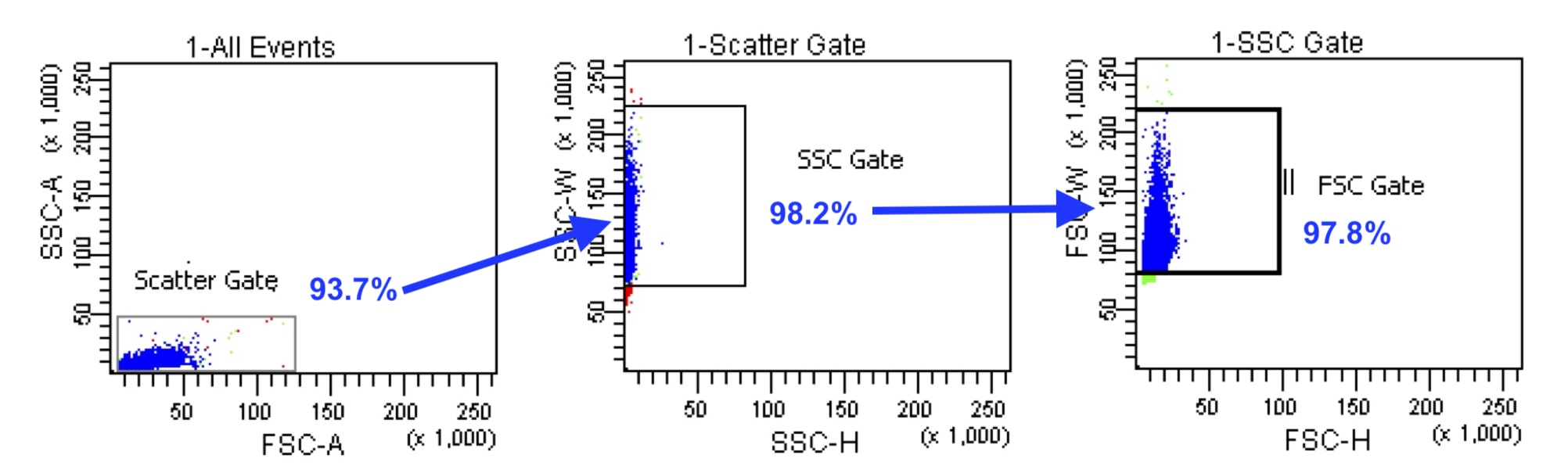

Supplement: S11 Fig — (TIF) [file pbio.2005594.s011.tif]
