# Supplementary material for: The mammalian decidual cell evolved from a cellular stress response
Source: PLoS Biol. 2018 Aug 24;16(8):e2005594. doi: 10.1371/journal.pbio.2005594 (PMC6108454; doi:10.1371/journal.pbio.2005594)
Supplement: S2 Table — (DOCX) [file pbio.2005594.s013.docx]

**Table S2**

| ***Gene*** | **Forward (5’-3’)** | **Reverse (5’-3’)** |
| --- | --- | --- |
| *CD45* | TTTAAGATGGACTTATAACAGGAATTTCG | GCGTTCATAATTAACAGAAGCATGG |
| *EBF4* | GATCTGTATGTTCGGCTCATCG | GGTCAGATGGTGTCTCATTTCG |
| *GATA2* | AGCGAGAGTCTCCTACAGC | CTTGGAGAAAGGGCTCACG |
| *HOXA10* | GTTCACACTTACCTCTGACTTCC | TACTCCCTCCTCTTCATAAGGC |
| *HOXA11* | GGCAACAATGAGGACAAATCC | TTGAGCATTCGGGAGAGC |
| *HOXD9* | CAACAGCAGCAACTTGACCCAA | AAATCCTAGCCACTTCGTAGC |
| *HOXB2* | CGCCTCTCTCTGCAGCTC | CCTGCAGTCCAGGGGTATC |
| *PGR* | AAGGAGAGTCTGCCACAGG | GCAACCAGATGCTTCATCCC |
| *CXCL12* | CTTGGTGACCTTGCTGGTG | TGTTGGCTTTGGCTACATTG |
| *GALNT15* | AGATCCGAATTGTGGAGACC | GGAGAGATTGGCCAGAAACC |
| *GPX3* | GCAGGAAAATACATCCTCTTCG | TTTCCAAATTGGTTGCAAGG |
| *IGFBP3* | AATTCCATCCACACCACACC | GACCTGTTGCTCCTGTTTGG |
| *MRGPRF* | GATCCATGCCCAGAACAGG | CACAGGCAGTTGAGCAGAAA |
| *PDK4* | AGAGCCTCATGGATTTGGTG | ACCTTGATCAGCGCGTCTAC |
| *ZBTB16* | CTTCGTCTGTGATCAGTGTGG | GAGTGCGCTCTGAGTCTGG |
| *FOXO1* | GGGTAACCTGTCCTATGCCG | TGCACTCGGATGAACTTGCT |
| *FOXO3* | CCCAGATCTATGACTGGATGG | GGTCCCTTCGTTTTGTACCC |
| *TBP* | CTCTTCCATTCACAGACTCTTACC | TCAAGTTTACAACCAAGATTCACG |
