# Supplementary material for: The mammalian decidual cell evolved from a cellular stress response
Source: PLoS Biol. 2018 Aug 24;16(8):e2005594. doi: 10.1371/journal.pbio.2005594 (PMC6108454; doi:10.1371/journal.pbio.2005594)
Supplement: S3 Table — siRNA, small interfering RNA (DOCX) [file pbio.2005594.s014.docx]

**Table S3.**

| **Name** | **Sense (5’-3’)** | **Antisense (5’-3’)** |
| --- | --- | --- |
| FOXO1 s671 | GGAAGAAUUCGAUUCGCCAUU[dT][dT] | AAUGGCGAAUCGAAUUCUUCC[dT][dT] |
| FOXO1 s1084 | GUCUAUCCUUCGUCCACCAUU[dT][dT] | AAUGGUGGACGAAGGAUAGAC[dT][dT] |
| FOXO3 s695 | GGCACAACUUGUCCCUACA[dT][dT] | UGUAGGGACAAGUUGUGCC[dT][dT] |
| FOXO3 s1844 | CUUUGUAUUCCACUAGCGU[dT][dT] | ACGCUAGUGGAAUACAAAG[dT][dT] |
| GR s1926 | CCAAUGUAAACAUAUGCUA[dT][dT] | UAGCAUAUGUUUACAUUGG[dT][dT] |
| GR s698 | CAGAUGAUCCAUUUCUAUU[dT][dT] | AAUAGAAAUGGAUCAUCUG[dT][dT] |
